# Non-linearity of Metabolic Pathways Critically Influences the Choice of Machine Learning Model

**DOI:** 10.3389/frai.2022.744755

**Published:** 2022-06-10

**Authors:** Ophélie Lo-Thong-Viramoutou, Philippe Charton, Xavier F. Cadet, Brigitte Grondin-Perez, Emma Saavedra, Cédric Damour, Frédéric Cadet

**Affiliations:** ^1^University of Paris, BIGR—Biologie Intégrée du Globule Rouge, Inserm, UMR_S1134, Paris, France; ^2^Laboratory of Excellence GR-Ex, Paris, France; ^3^Laboratory DSIMB, UMR_S1134, BIGR, Inserm, Faculty of Sciences and Technology, University of La Reunion, Saint-Denis, France; ^4^PEACCEL, Artificial Intelligence Department, Paris, France; ^5^EnergyLab, EA 4079, Faculty of Sciences and Technology, University of La Reunion, Saint-Denis, France; ^6^Departamento de Bioquímica, Instituto Nacional de Cardiología Ignacio Chávez, Mexico City, Mexico

**Keywords:** artificial intelligence, machine learning, non-linear modeling, drug target identification, *Trypanosoma cruzi* detoxification pathway, *Entamoeba histolytica* glycolysis pathway, penicillin production

## Abstract

The use of machine learning (ML) in life sciences has gained wide interest over the past years, as it speeds up the development of high performing models. Important modeling tools in biology have proven their worth for pathway design, such as mechanistic models and metabolic networks, as they allow better understanding of mechanisms involved in the functioning of organisms. However, little has been done on the use of ML to model metabolic pathways, and the degree of non-linearity associated with them is not clear. Here, we report the construction of different metabolic pathways with several linear and non-linear ML models. Different types of data are used; they lead to the prediction of important biological data, such as pathway flux and final product concentration. A comparison reveals that the data features impact model performance and highlight the effectiveness of non-linear models (e.g., QRF: RMSE = 0.021 nmol·min^−1^ and R^2^ = 1 vs. Bayesian GLM: RMSE = 1.379 nmol·min^−1^ R^2^ = 0.823). It turns out that the greater the degree of non-linearity of the pathway, the better suited a non-linear model will be. Therefore, a decision-making support for pathway modeling is established. These findings generally support the hypothesis that non-linear aspects predominate within the metabolic pathways. This must be taken into account when devising possible applications of these pathways for the identification of biomarkers of diseases (e.g., infections, cancer, neurodegenerative diseases) or the optimization of industrial production processes.

## Introduction

**Figure 1:**
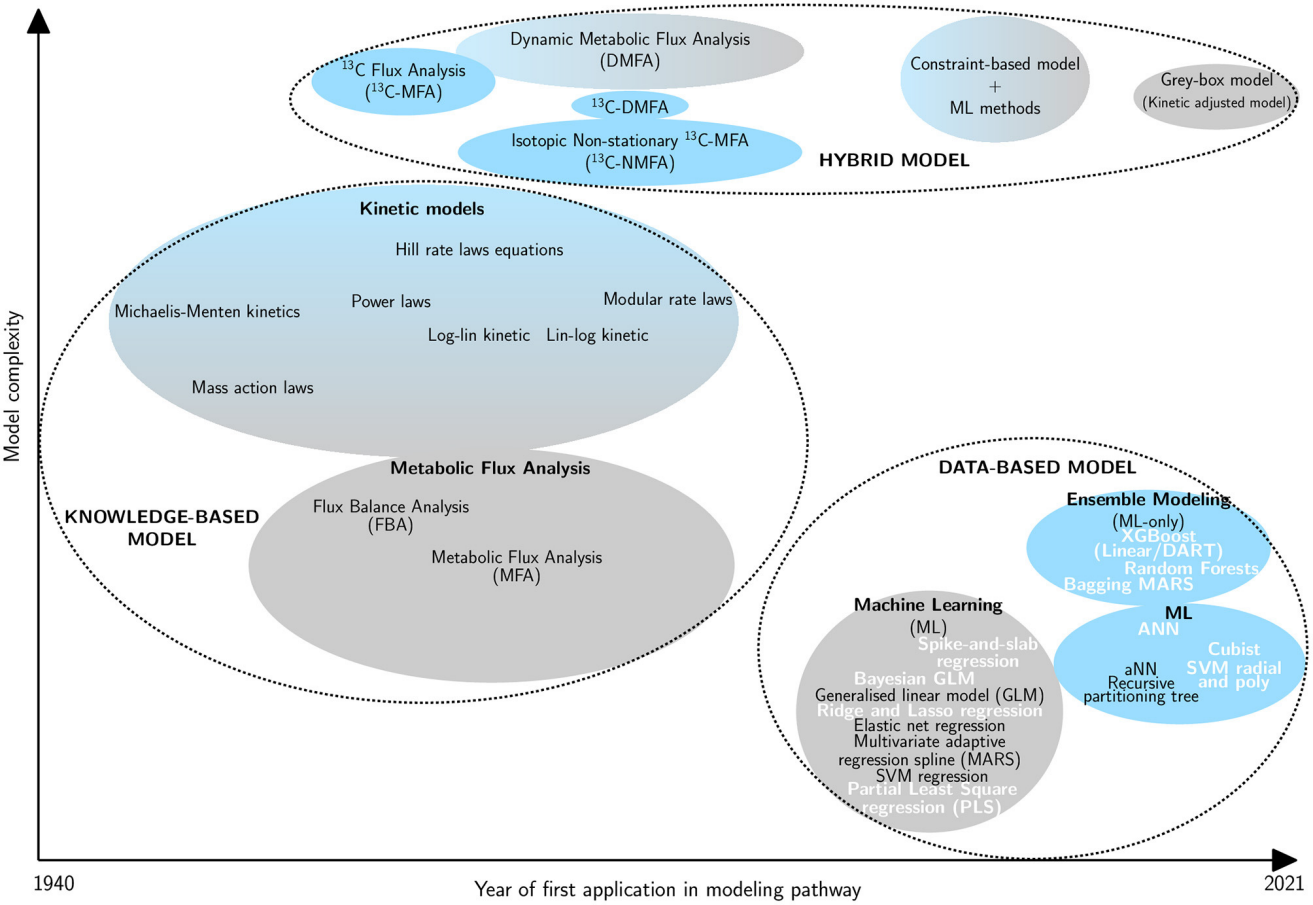
Classification of metabolic pathway modeling methods according to their complexity and the year of first application in this field. The ellipse size is proportional to the occurrence of the method for pathway modeling in the literature. Three main groups are defined: knowledge-based model (Michaelis and Menten, 1913; Chance, 1943; Shapiro and Shapley, 1965; Garfinkel et al., 1970; Savageau, 1970, 1988; Fell and Small, 1986; Hatzimanikatis and Bailey, 1997; Curto et al., 1998; Heijnen, 2005; Liebermeister et al., 2010), data-based model (Wu et al., 2016; Cuperlovic-Culf, 2018; Ajjolli Nagaraja et al., 2019; Zampieri et al., 2019; Zhang et al., 2019; Kim et al., 2020) and hybrid model (Wiechert et al., 1997; Drysch et al., 2003; Antoniewicz et al., 2007; Nöh et al., 2007; Leighty and Antoniewicz, 2011; Antoniewicz, 2015; Pan et al., 2017; Yousoff et al., 2017; Heckmann, 2018; Oyetunde et al., 2019; Zampieri et al., 2019; Lo-Thong et al., 2020; Rana et al., 2020). Linear methods are represented in gray and non-linear ones are in blue. Methods in bold and white are those evaluated in this study.

Machine learning (ML) holds an increasingly prominent place in the field of biology. Indeed, it can lead to better results and has a large range of applications including: drug design using machine leaning algorithms such as the support vector machine (SVM) algorithm to perform structure-activity relationship analysis (Hartwell et al., 1999; Burbidge et al., 2001; Réda et al., 2020); directed protein evolution and enzyme function prediction (Li et al., 2018; Wu et al., 2019); reconstruction of biological routes (Kotera et al., 2013; Baranwal et al., 2020) or modeling and optimization of metabolic pathways (Zhang et al., 2019; Kim et al., 2020). With regard to the latter topic, several methods have been developed to analyze complex biological systems (Figure 1):

- The **knowledge-based model** including kinetic models (Chance, 1943; Sel'Kov, 1968; Curto et al., 1997, 1998; Hatzimanikatis et al., 1998; Visser and Heijnen, 2003; Liebermeister et al., 2010) and metabolic flux analysis methods (Fell and Small, 1986; Stephanopoulos, 1999);
- The **data-based model** including ML algorithms and ensemble learning (Zelezniak et al., 2018; Ajjolli Nagaraja et al., 2019; Oyetunde et al., 2019);
- The **hybrid model** including combinations of models or modified preceding methods (Cascante et al., 2002; Morgan and Rhodes, 2002).

Although, these analyses are conducted on metabolic pathways, few of them are used to predict their fluxes. Among these few works on metabolic fluxes, it is interesting to highlight those of (Ajjolli Nagaraja et al., 2019). For the present work, the method of greatest interest is the data-based model and more precisely, ML. In fact, ML abounds in various methods and is a promising and growing approach that could greatly help to improve existing models, integrate multi-omics data and save researchers' time. Also, a distinction can be made between ML methods: some are linear (ridge and lasso regression, multivariate adaptive regression spline…) and others are non-linear (artificial neural network, k-nearest neighbors, decision tree…). In addition, the non-linearity of metabolic pathway is considered inherent to the pathway, depending on the non-linearity of chemical reaction kinetics and that related to regulatory processes (Song and Ramkrishna, 2013; Yasemi and Jolicoeur, 2021). Reviews on the fundamentals of Metabolic Control Analysis (Heinrich and Rapoport, 1974; Kacser et al., 1995) suggest mathematically that the pathway fluxes are non-linear. Moreover, experiments were done on glycolytic fluxes cells, where intact cells were incubated at different glucose concentrations (Marín-Hernández et al., 2020). The results showed a clearly hyperbolic behavior of the experimental data. Another experimental data used notably in this study indicates that the pattern is non-linear (Moreno-Sánchez et al., 2008; González-Chávez et al., 2015). These experimental data demonstrate that the pathway fluxes are non-linear. However, it has not yet been investigated whether linear or non-linear methods are more efficient in predicting pathway fluxes, and how to choose the appropriate one.

**Figure 2:**
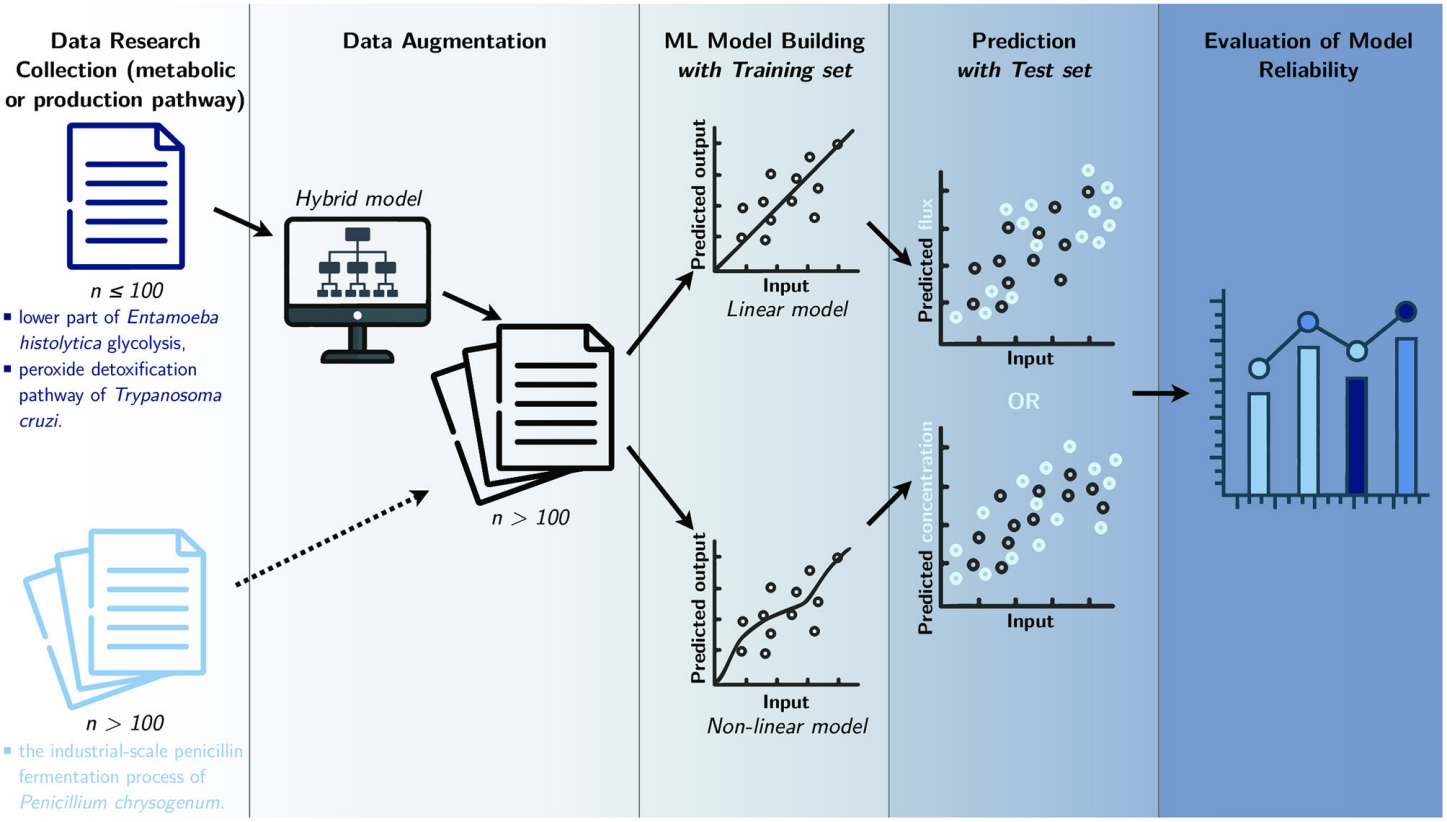
Study workflow. Data from three different metabolic pathways are collected and used to build data-based models. Datasets that contain a small amount of data (*n*) go through a process of data augmentation, before being separated into two sets: training set and test set. Then, in order to predict the final flux or final product concentration, multiple ML models are built with the training set, while the test set is used to assess the final models. The resulting predictions are compared in a last step to evaluate model reliability.

**Figure 3:**
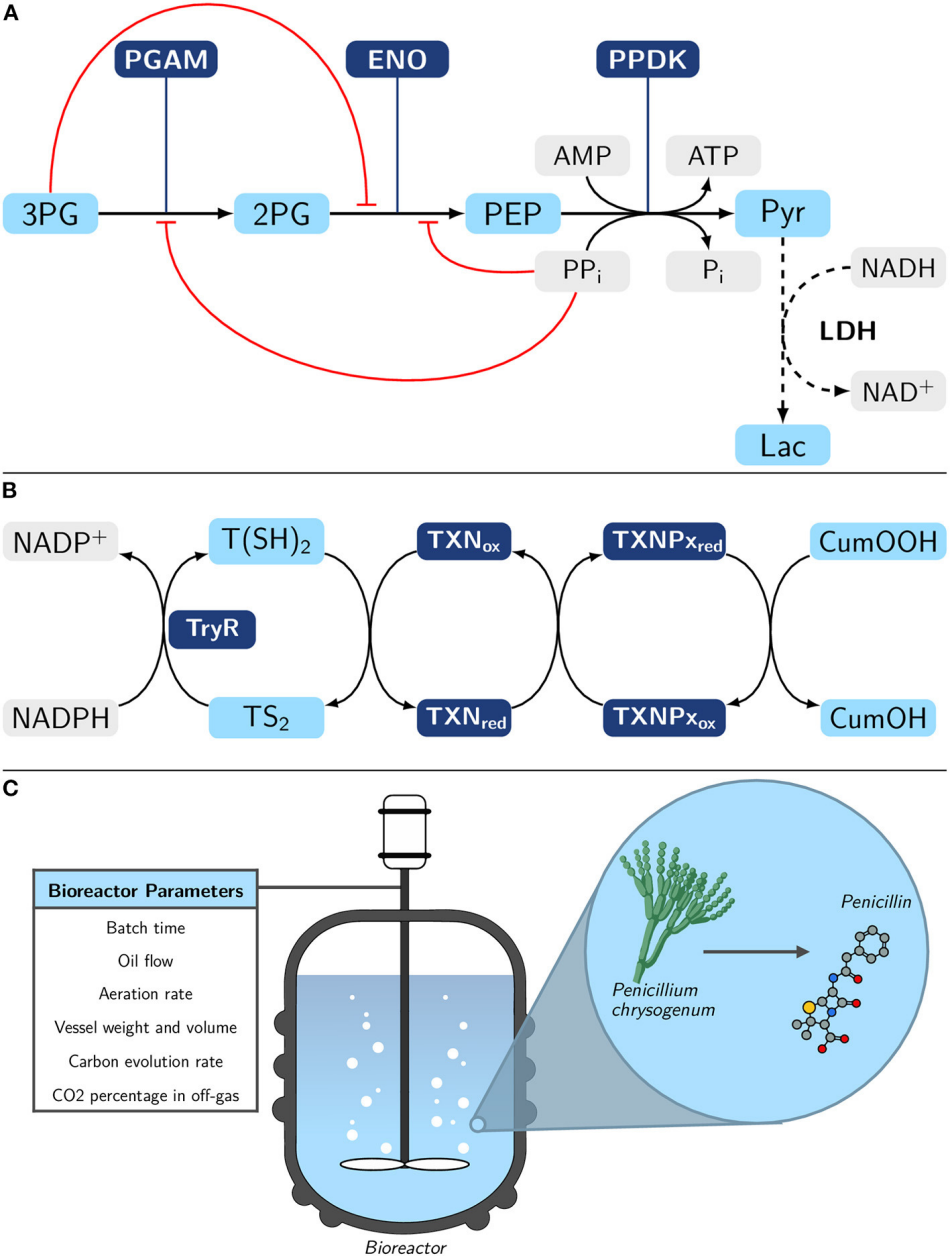
Overview of the three metabolic pathways modeled with machine learning methods. **(A)** Lower part of *E. histolytica* glycolysis pathway with pyruvate (Pyr) formation from 3- phosphoglycerate (3PG). The L-lactate (Lac) formation (dashed lines) is not part of the natural pathway; however, lactate dehydrogenase (LDH) has been added in order to experimentally follow the final flux and establish a quasi-steady-state to Lac (Moreno-Sánchez et al., 2008). Metabolite inhibitions are represented in red. PGAM, 3-phosphoglycerate mutase; 2PG, 2-phosphoglycerate; ENO, enolase; PEP, phosphoenolpyruvate; PPDK, pyruvate phosphate dikinase. **(B)** Tryparedoxin-dependent hydroperoxide detoxification pathway in *Trypanosoma cruzi* (González-Chávez et al., 2015). Reduction of cumene hydroperoxide (CumOOH) is assessed here. TryR, trypanothione reductase; T(SH)_2_, trypanothione; TS_2_, trypanothione disulfide; TXN_ox/red_, oxidized/reduced tryparedoxin; TXNPx_ox/red_, oxidized/reduced tryparedoxin peroxidase. **(C)** Simplified representation of the industrial-scale penicillin fermentation process of *Penicillium chrysogenum*. The bioreactor parameters represented here are those that will be of interest in this study. See a more detailed scheme in the work of Goldrick et al. (2015). Experimental details for **(A,B)** are provided in section Material and Methods.

Therefore, this study aims to elucidate the most appropriate methods to model three distinct metabolic pathways by designing and comparing five linear and eight non-linear machine learning-based methods (Figure 2):

- The lower part of *Entamoeba histolytica* glycolysis (Figure 3A), one of the major metabolic pathways of the parasite (Moreno-Sánchez et al., 2008; Muller et al., 2012; Pineda et al., 2015), through the use of a recently developed model (Lo-Thong et al., 2020);
- The peroxide detoxification pathway of *Trypanosoma cruzi* (Figure 3B) (González-Chávez et al., 2015, 2019);
- The industrial-scale penicillin fermentation process of *Penicillium chrysogenum* (Figure 3C) (Goldrick et al., 2015).

Although these machine-learning approaches have been used to model metabolic pathways, few studies have focused on their usefulness in predicting flux (Wu et al., 2016; Ajjolli Nagaraja et al., 2019).

Creating an efficient ML model depends on the availability of a large amount of experimental data (L'Heureux et al., 2017; Schmidt et al., 2019). The measurement of fluxes is cumbersome to carry out experimentally and hinders the possibility of having massive data. Because of the scarcity of these large experimental datasets in the literature, the methodology employed here consists of applying data augmentation to the first two pathways by using hybrid models (Figure 2). These hybrid models, called gray-box models, often predict better results than pure knowledge-based models or data-based models (Wei et al., 2018; Lo-Thong et al., 2020; Pintelas et al., 2020); in this study, the gray-box models consist of metabolic networks that include an adjustment term in one or more kinetic equations.

In this study, models are based both on experimental datasets and predicted data coming from the previous gray-box model. Here, we show that random forest models are the most effective, with a high predictive capacity starting from predicted and experimental enzyme activities or experimental parameters collected from a bioreactor. Also, two other models stand out as good ways to predict the flux or the final product concentration: XGBoost Linear and Cubist models. This shows the importance of using a non-linear model to design metabolic pathways. Based on these findings, we propose a means of decision support for researchers who wish to use machine learning techniques as a starting or a complementary method for modeling and for improving existing biological pathway models. By greatly increasing the quality of the outputs (flux prediction), machine learning opens the way to better drug target identification within a pathway, efficient disease modeling at molecular level and more efficient optimization for industrial production of metabolites.

## Materials and Methods

### Experimental Procedures

The lower part of glycolysis is reconstituted *in-vitro* in a reaction assay medium described in a recent work (Moreno-Sánchez et al., 2008), containing different recombinant enzymes (PGAM, ENO and PPDK). The reaction was started by adding 3PG (4 mM). An additional reaction is added, the formation of lactate with lactate dehydrogenase (Figure 3A), in order to follow the flux of the overall pathway by following the rate of NADH oxidation, for more details, see Moreno-Sánchez et al. (2008) works. Concerning the peroxide detoxification pathway (Figure 3B), each enzyme was individually titrated, while keeping the other parameters in the *in-vitro* system constant. The pathway flux was determined in parallel by observing NADPH oxidation, see González-Chávez et al. (2015) for more information. Finally, the experimental procedures that were followed to obtain penicillin production data are described in the studies of Goldrick et al. (2015).

### Lower Part of Glycolysis Datasets

Two datasets are constructed here by applying data augmentation, using a gray-box model detailed in one of the following sections. For the first one, an exploration around the experimental data flux (43 ± 10 nmol·min^−1^) from Moreno-Sánchez et al. (2008) at pH 6 is conducted. In fact, a sample of 2,000 normally distributed enzymatic balances was generated with the *sample* function on RStudio and resulted in a predicted flux between 0 and 53 nmol·min^−1^ with the gray-box model. The term balance refers to a set of activities of the enzymes involved in the cascade of reactions. The second dataset is made up of experimental and predicted (gray-box model) data of PGAM, ENO and PPDK activities and pathway flux (J). The experimental data are obtained from plots of Moreno-Sánchez study (Moreno-Sánchez et al., 2008) (only the dots), while the predicted data are obtained with the gray-box model developed in a recent work (Lo-Thong et al., 2020), by varying each enzyme activity from 0 to 1000 mU with a step of 25 mU. These datasets are shown in Supplementary Tables 7, 8 respectively.

### Peroxide Detoxification Datasets

The second studied pathway consisted first of 58 experimental enzymatic balances and their corresponding flux. After applying data augmentation by using a gray-box model of this pathway, a bigger dataset of 1,671 data was obtained. As with the previous dataset, a combination of data normally distributed is generated with the *sample* function on RStudio, resulting in a predicted flux ranging from 0 to 11.46 nmol·min^−1^. The new dataset is a mix of the previous experimental data and new predicted data of enzyme activities (TryR, TXN and TXNPx); final flux and is shown in Supplementary Table 9.

### The Gray-Box Models

The two following pathways are modeled with an open-source software called COPASI (Version 4.24) (Hoops et al., 2006): the second part of glycolysis and the peroxide detoxification pathway. This software is used for metabolic network design, analysis and optimization. The first gray-box model, representing the lower part of glycolysis, is taken from Lo-Thong et al. (2020) work. It is based on the use of enzyme properties, including kinetic parameters and kinetic equations. To enhance the flux predictions, they suggested adding an adjustment term to the PPDK kinetic equation. The whole process concerning the composition of this term is explained in the previous work (see Methodology part of Lo-Thong et al., 2020 and Supplementary Table 1).

The second gray-box model represents the peroxide detoxification pathway and is built specifically for this study. It contains kinetic parameters and equations of three enzymes: TryR, TXN and TXNPx (Table 1). Also, we proposed to add two adjustment terms in TryR and TXNPx equations to improve flux predictions (Table 1). These are determined in the same way as the terms used for the glycolysis pathway. In fact, a first model was provided by González-Chávez et al. (2019) and could predict the final flux quite well when TryR and TXN activities were varied. However, it overestimated the flux when TryR activity was varied and underestimated it when TXNPx activity was varied. Therefore, we suggest adding a first adjustment term **α**(***V***_***f***_ **−** ***V***_***f*****0**_) in order to increase TryR rate and a second adjustment term **β**(***V***_***f***_ **−** ***V***_***f*****0**_**)** to decrease TXNPx rate. In these adjustment terms, α and β are defined numbers selected as the best for flux prediction from a tested range, *V*_*f*_ is TryR (or TXNPx) maximum rate in the forward direction in the model and *V*_*f*0_ TryR (or TXNPx) maximum rate in the forward direction used in the *in vitro* reconstitution. Also, as *V*_*f*_ of TryR (or TXNPx) is equal to *V*_*f*0_ when TXN's/TXNPx's (or TryR's/TXN's) activity is varied, we multiplied α (or β) by *V*_*f*_ − *V*_*f*0_, so that the adjustment term would be zero when *V*_*f*_ = *V*_*f*0_ and the flux predictions are not modified in these cases mentioned above.

**Table 1:** Kinetic equations used in the gray-box model of the peroxide detoxification pathway (González-Chávez et al., 2015).

**Enzyme**	**Kinetic equations**
TryR^a^	$v=\frac{V_{f}\frac{AB}{K_{mA}K_{mB}}-V_{r}\frac{PQ}{K_{mP}K_{mQ}}+\alpha \left(V_{f}-V_{f0}\right)}{1+\frac{A}{K_{mA}}+\frac{B}{K_{mB}}+\frac{P}{K_{mP}}+\frac{Q}{K_{mQ}}+\frac{AB}{K_{mA}K_{mB}}+\frac{AP}{K_{mA}K_{mP}}+\frac{BQ}{K_{mB}K_{mQ}}+\frac{PQ}{K_{mP}K_{mQ}}+\frac{ABP}{K_{mA}K_{mB}K_{mP}}+\frac{BPQ}{K_{mB}K_{mP}K_{mQ}}}$
TXN^b^	$v=\frac{V_{f}\left(AB-\frac{PQ}{K_{eq}}\right)}{AB+K_{mB}A+K_{mA}B\left(1+\frac{Q}{K_{iQ}}\right)+\frac{V_{f}}{V_{r}K_{eq}}\left[K_{mQ}P\left(1+\frac{A}{K_{iA}}\right)+Q\left(K_{mP}+P\right)\right]}$
TXNPx^c^	$v=\frac{V_{f}\left[CumOOH\right]\left[TXN_{red}\right]+\beta \left(V_{f}-V_{f0}\right)}{K_{mTXN_{red}}\left[CumOOH\right]+K_{mCumOOH}\left[TXN_{red}\right]+\left[CumOOH\right]\left[TXN_{red}\right]}$

a *A, B and K_mA_, K_mB_ are respectively the concentrations and K_m_ of the substrates NADPH and TS_2_; P, Q and K_mP_, K_mQ_ are the concentrations and K_m_ of the products NADP^+^ and T(SH)_2_; α(V_f_ − V_f0_) is the adjustment term with α, a defined number, V_f0_, TryR maximum rate in the forward direction used in the in vitro reconstitution and V_f_ is TryR maximum rate in the forward direction in the model*.

b *A, B and K_mA_, K_mB_ are respectively the concentrations and K_m_ of the substrates T(SH)_2_ and TXN_ox_; P, Q and K_mP_, K_mQ_ are the concentrations and K_m_ of the products TS_2_ and TXN_red_*.

c *$\beta \left(V_{f}-V_{f0}\right)$ is the adjustment term with β, a defined number, V_f0_, TXNPx maximum rate in the forward direction used in the in vitro reconstitution and V_f_ is TXNPx maximum rate in the forward direction in the model*.

Also, residual values are determined to evaluate how accurate the gray-box model is, and calculated as follows (1):


(1) $$\begin{array}{l} e\;=\;y\;-\;\hat{y} \end{array}$$

where *e* is the residual, *y* is the observed value and ŷ the corresponding predicted value.

### Data Augmentation

For the datasets with <100 data, a process called data augmentation is performed. It consists of using models that accurately predict the experimental data to generate a new bigger dataset. Two different gray-box models are used in this study for the lower part of glycolysis pathway, retrieved from a recent study (Lo-Thong et al., 2020), and for the peroxide detoxification pathway (built for the present work). The gray-box models built on COPASI is set up to predict the variation of the final product concentration over the first hour for a given set of enzyme activities; then the COPASI outputs are processed to obtain the final flux of the studied metabolic pathway. Also, the overall process from the one-hour simulation for each enzymatic balances to the determination of the final flux is then automatized and applied to a range of enzymatic balances detailed in the previous subparts (Lower Part of Glycolysis Datasets and Peroxide Detoxification Datasets).

### Dataset Analysis and Non-linearity Assessment

A brief analysis of the datasets is performed, including an examination of data distribution and the calculation of linear correlations between the input and output variables.

The determination of linear correlation between the inputs and output variables allows the assessment of the non-linearity for each studied metabolic pathway. As a rule of thumb, we consider that the non-linearity is high when one or more inputs has a linear correlation lower than 0.6. The lower the linear correlation, the greater the degree of non-linearity of the pathway.

### Machine Learning Models Building and Selection

To model the metabolic pathway, different machine learning models are developed on RStudio (Version 1.2.5001), with the help of Classification And Regression Training (caret, Version 6.0-86) (Kuhn, 2020).

The datasets are split into 80/20 for the training and test sets, and a k-fold cross-validation (with k = 10 for Dataset 1, 2 and k = 3 for Dataset 3) is performed on the models with the training set.

After this, the best models are selected based on:

The root-mean-square error (RMSE):


(2) $$\begin{array}{l} RMSE\;=\;\sqrt{\frac{1}{n}\overset{n}{\underset{i\;=1}{\sum }}{\left({\hat{Y}}_{i}-Y_{i}\right)}^{2}} \end{array}$$

with *Y*_*i*_ and *Ŷ*_*i*_ being respectively the observed and predicted values, n being the total number of values and i = 1, 2…*n*;

the coefficient of determination (R^2^):


(3) $$\begin{array}{l} R^{2}=\;1-\;\frac{\sum_{i=1}^{n}{\left(Y_{i}-{\hat{Y}}_{i}\right)}^{2}}{\sum_{i=1}^{n}{(Y_{i}-\hat{Y})}^{2}} \end{array}$$

with *Y*_*i*_ and *Ŷ*_*i*_ respectively the observed and predicted values, *n* being the total number of values and i = 1, 2…*n*.

Also, a calculator was used for modeling the metabolic pathways, which has the following characteristics: cluster 2x Intel Xeon E5-2630v4 Broadwell-EP @ 2.20GHz 10 cores, 8x 16GB of RAM, 2400MHz, DDR4, ECC.

## Results

As previously mentioned, ML models could have different applications in biology, including the identification of biomarkers, i.e., a valuable, quantitative component (metabolites, proteins, enzymes…), within a metabolic pathway for health purposes (diseases diagnosis, treatment) or the optimization of a valuable production pathway. Therefore, we have targeted three different datasets based on these two applications. The first one concerns the lower part of glycolysis in *Entamoeba histolytica* (Figure 3A) and contains a set of enzyme activities for which the final flux has been measured (Moreno-Sánchez et al., 2008). The second pathway is the tryparedoxin-dependent hydroperoxide detoxification pathway in *Trypanosoma cruzi* (Figure 3B), which provides the same type of data as in the previous dataset (González-Chávez et al., 2015). It is important to consider how essential these two previous pathways are, as they play a significant role in the survival of these parasites. Given the small size of the experimental dataset, we use two gray-box models: one developed recently (Lo-Thong et al., 2020) and the other developed in this study, to generate a larger dataset for these two pathways (Datasets 1 and 2) before building the ML models (Figure 2).

The last metabolic pathway modeled here is the penicillin fermentation process in *Penicillium chrysogenum* (Figure 3C). This dataset did not need to be enlarged (Dataset 3), and we used it to build different ML models (Figure 2).

### Example 1: The Lower Part of *Entamoeba histolytica* Glycolysis

#### The Gray-Box Model Allows the Building of Huge Datasets

Since the amount of experimental data is limited, the first step here is to build a robust model to generate more data.

As explained in the Methods section, the gray-box model developed in a previous work contains all kinetic parameters and kinetic equations of PGAM, ENO and PPDK (Lo-Thong et al., 2020). In order to improve the flux prediction, the first two enzymes employ the Michaelis-Menten reversible rate equation, whereas the third employs a modified termolecular reaction reversible rate equation including an adjustment term in the denominator (Supplementary Table 1). The resulting fluxes show good reliability of the model to predict the final experimental flux (R^2^ ≈ 0.95 and RMSE = 1.993 nmol·min^−1^), even when enzyme activities are varied (Supplementary Figures 1A-C).

The calculation of residuals shows a defined pattern that is the same for PGAM and ENO. It reveals a general trend of the model to underestimate the flux for low enzyme activity values, and overestimate it for high enzyme activity values (Supplementary Figures 1D,E). Concerning PPDK, the gray-box model tends instead to underestimate the final flux when the enzyme activity is varied, with an exception for the last point (at 232.13 mU), which is overestimated (Supplementary Figure 1F). The model is quite accurate to predict the pathway flux and presents low residuals between−3.4-4.7 nmol·min^−1^.

The next step of this work consists of using the *in-silico* model for generating larger datasets, a process we call data augmentation. The first new dataset contains 2,000 enzyme balances evolving around the experimental ones (see Supplementary Table 6, Supplementary Figure 2). The term balance refers to a set of concentrations of the enzymes involved in the cascade of reactions. The predicted final fluxes vary between 0 and 60.84 nmol·min^−1^; the distribution of the other data from the first dataset is shown in Supplementary Figure 3. In fact, the predicted fluxes count with the highest representation are within the experimental data of the reconstituted pathway (Moreno-Sánchez et al., 2008) and *in vivo* pathway fluxes in live parasites (Pineda et al., 2015). In order to compare the models, a second dataset (Dataset 1) is generated and includes 68,950 data for which all enzyme activity is varied between 0 and 1,000 mU (see Supplementary Tables 2, 7). The final fluxes are then predicted and fluctuate between 0 and 215.45 nmol·min^−1^; additional information is provided in Supplementary Table 2, Supplementary Figure 4.

**Figure 4:**
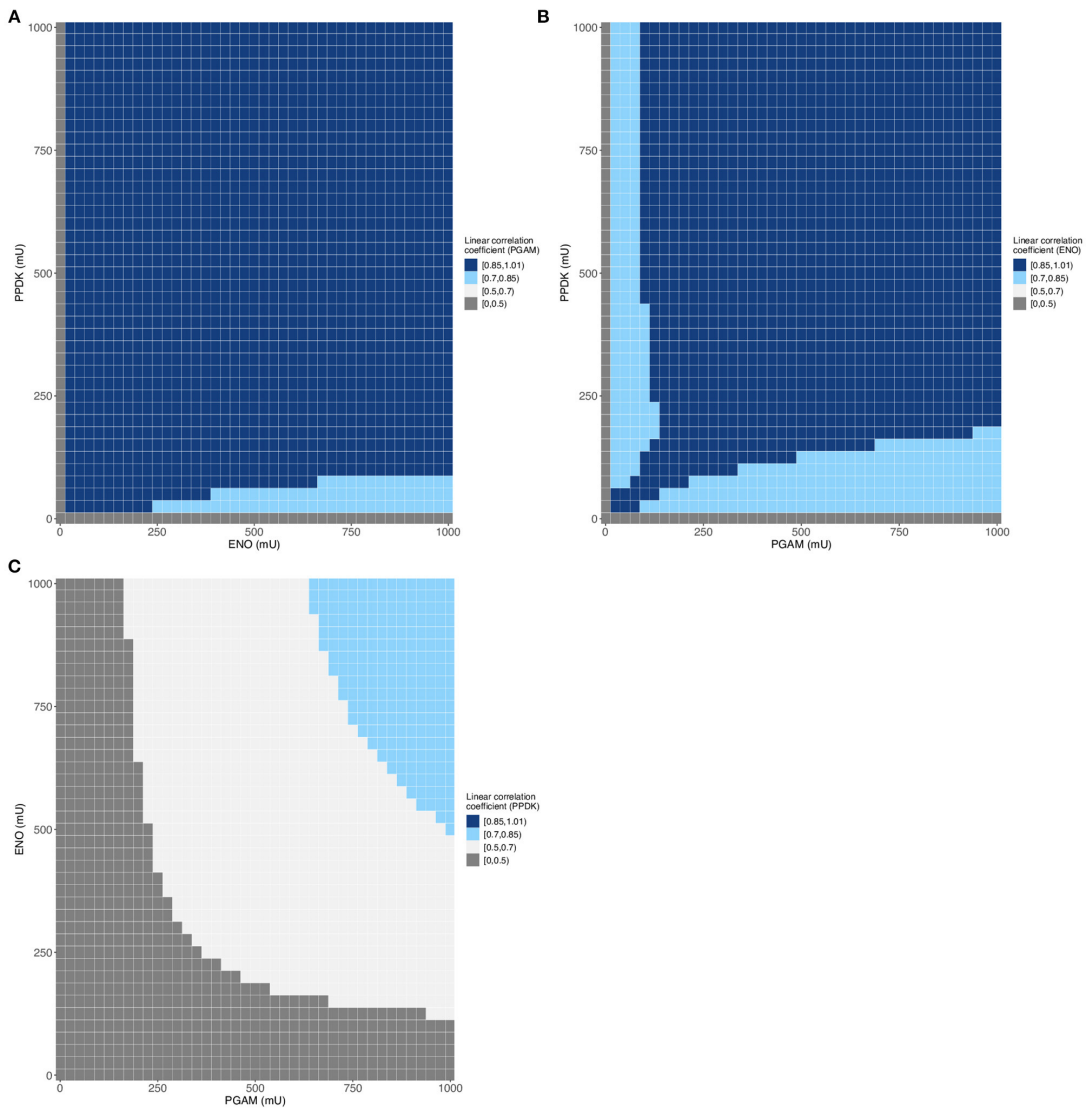
Evolution of linear correlation coefficient for each enzyme of Dataset 1 (68,950 data). **(A–C)** Variation of PGAM **(A)**, ENO **(B)** or PPDK **(C)** correlation coefficient between enzymes activities and the predicted final flux.

We then plotted the final flux in function of the enzyme activity for the largest dataset (Supplementary Table 7) and obtained the same type of curve as we did previously (Supplementary Figures 2, 5). Indeed, variations of PGAM activity have a great impact on the final flux, while those of ENO and PPDK have a lesser impact on the pathway flux (Supplementary Figure 5). It should also be noticed that the experimental fluxes are in the lower part of the predicted flux values. The insets show a gap between the experimental flux values and the dataset flux values; this difference is due to the intervals between two values, used in the two cases, with the interval being smaller for the experimental dots (7–85 mU) than for the predicted data (25 mU). Following this initial analysis of the data, we assessed the correlation between the various variables. The table of correlation shows that the enzymes and the final flux are correlated to varying degrees, with the highest correlation coefficient for PGAM, followed by ENO, and the lowest coefficient for PPDK (Table 2). These linear correlation coefficients provide insight into the degree of non-linearity of this metabolic pathway. Here, only PPDK has a linear coefficient lower than 0.6 indicating that the lower part of glycolysis has a large degree of non-linearity. Also, even if the mean value of the correlations is above 0.5 (Table 2), we observe a weak linear correlation for many ranges of enzyme activity (Figure 4) when one of the enzymes is varied over the three, for example for PPDK when PGAM varies between 0 and 625 mU and ENO between 0 and 1,000 mU (Figure 4C). These results indicate significant non-linearity in the metabolic pathway, particularly for PPDK and ENO. In addition, these results lead to the same conclusions as those from flux control coefficient calculations (Lo-Thong et al., 2020): the enzyme exerting the greatest flux control is PGAM, followed by ENO, and PPDK has the weakest control of the pathway flux.

**Table 2:** Table of mean linear correlations between the enzyme activities and the predicted final flux (*J*_*pred*_) for Dataset 1.

	***J_pred_***
PGAM	0.90
ENO	0.85
PPDK	0.53

Good quality augmented datasets having been generated; they are used to test different ML approaches in the following section.

#### Non-linear Machine Learning Methods for Metabolic Pathway Modeling Outperform Rborist

**Figure 5:**
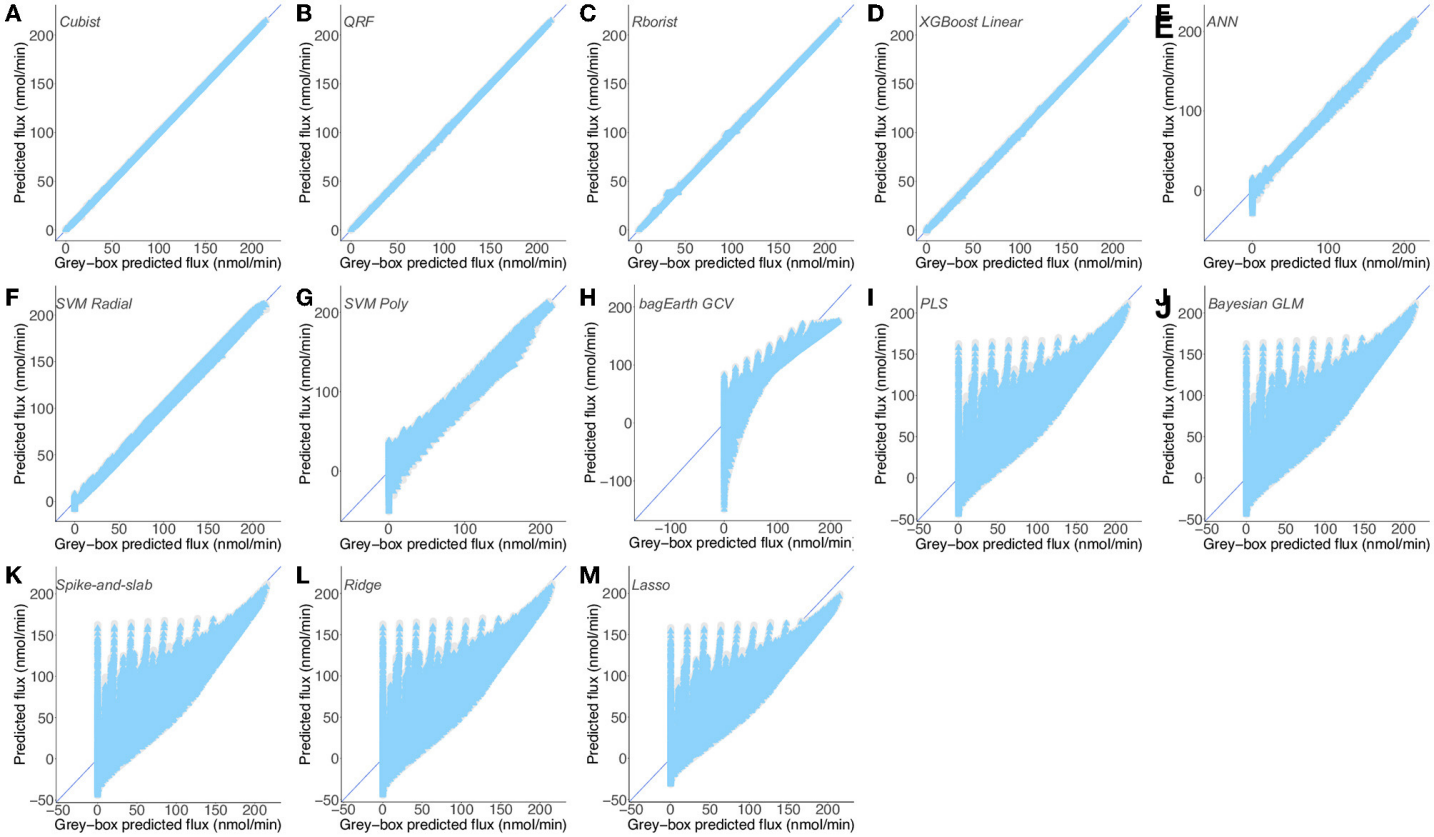
Predictions of a mix of experimental and gray-box predicted flux by different predictive models. **(A–D)** Flux from Dataset 1 (Supplementary Table 7) predicted by the Cubist **(A)**, QRF **(B)**, Rborist **(C)**, XGBoost Linear **(D)**, ANN **(E)**, SVM Radial **(F)**, SVM Poly **(G)**, bagEarth GCV **(H)**, PLS **(I)**, Bayesian GLM **(J)**, Spike-and-slab **(K)**, Ridge **(L)** and Lasso **(M)** models. Gray circles: training set and blue triangles: test set. See Table 3 for the statistical measurements of each model.

Based on the preceding data, we also investigate whether we can build a good predictive model by using linear and non-linear ML methods. In the study cited previously, Artificial Neural Networks (ANN) were used to predict the flux (Lo-Thong et al., 2020). Here, only one ANN model is developed and proves to be one of the best models obtained (Table 3 and Figure 5E). Among the designed models and for the first dataset (Supplementary Table 7), the random forest models stand out, with better flux prediction for the training set with the model built with Rborist package: cvRMSE = 0.883 nmol·min^−1^ and cvR^2^ = 0.995, than the QRF model: cvRMSE = 0.931 nmol·min^−1^ and cvR^2^ =0.994 (Supplementary Table 3, Supplementary Figures 6B,D). As for the test set, the QRF model outperforms the Rborist model, with RMSE = 0.076 nmol·min^−1^ and R^2^ = 1. Another good model, also non-linear, is the XGBoost Linear method, with cvRMSE = 0.833 nmol·min^−1^ and cvR^2^ = 0.995 (Supplementary Table 3, Supplementary Figure 6A). Moreover, the results obtained with Bayesian GLM, Lasso, Ridge, Spike-and-slab and the PLS model indicate that a linear model is not really adequate to describe this metabolic pathway. In fact, the PLS model gives the highest value for cvRMSE and the lowest value for cvR^2^ (Supplementary Table 3); also, we can see that the flux predictions are not very good (Supplementary Figure 6M). For the second dataset (Supplementary Table 7), we obtained almost the same results: first with the Cubist model (cvRMSE = 0.215 nmol·min^−1^ and cvR^2^ =1), then the two random forest models (Table 3). This time, better results are obtained with the QRF model: cvRMSE = 0.572 nmol·min^−1^ and cvR^2^ = 1, than with the Rborist model: cvRMSE = 0.647 nmol·min^−1^ and cvR^2^ = 1 for the training set (Table 3 and Figures 5A–C). The XGBoost Linear method also gives good flux predictions, with cvRMSE = 0.489 nmol·min^−1^ and cvR^2^ = 1 (Table 3 and Figure 5D). If the SVM Radial method gives almost good results (Table 3 and Figure 5F), it is no longer the case for the last two non-linear models (SVM Poly and bagEarth GCV) which present worse results in predicting flux, with much higher RMSE (Table 3 and Figures 5G,H).

**Table 3:** Summary table of statistical measurements for each predictive model.

	**Dataset 1**				**Dataset 2**				**Dataset 3**			
	**Training set**		**Test set**		**Training set**		**Test set**		**Training set**		**Test set**	
**Model**	**cvRMSE**	**cvR^**2**^**	**RMSE**	**R^**2**^**	**cvRMSE**	**cvR^**2**^**	**RMSE**	**R^**2**^**	**cvRMSE**	**cvR^**2**^**	**RMSE**	**R^**2**^**
**QRF (RF)**	0.572	1	0.218	1	0.183	0.996	0.022	1	0.814	0.993	0.134	1
**XGBoost Linear**	0.489	1	0.425	1	0.152	0.997	0.024	1	1.344	0.982	1.097	0.988
**Cubist**	0.215	1	0.154	1	0.128	0.998	0.057	1	1.22	0.985	1.224	0.985
**Rborist (RF)**	0.647	1	0.406	1	0.186	0.996	0.068	1	0.877	0.992	0.319	0.999
**ANN**	2.787	0.997	2.7	0.998	0.133	0.998	0.098	0.999	1.924	0.962	1.9	0.964
SVM Radial	3.373	0.996	3.36	0.996	0.349	0.989	0.233	0.996	1.897	0.964	1.902	0.964
SVM Poly	9.486	0.971	9.467	0.97	0.473	0.979	0.409	0.985	2.102	0.955	2.111	0.955
bagEarth GCV (bagging MARS)	20.893	0.858	22.2	0.844	0.956	0.916	0.964	0.914	2.384	0.942	2.418	0.941
Bayesian GLM	30.246	0.701	29.31	0.716	1.44	0.805	1.379	0.823	3.522	0.874	3.579	0.87
Spike-and-slab	30.246	0.701	29.31	0.716	1.44	0.805	1.379	0.823	3.522	0.874	3.579	0.87
Ridge	30.246	0.701	29.311	0.716	1.44	0.805	1.381	0.823	3.522	0.874	3.579	0.87
Lasso	30.567	0.701	29.518	0.714	1.462	0.803	1.407	0.821	3.526	0.874	3.582	0.87
PLS	30.246	0.701	29.309	0.716	1.581	0.765	1.55	0.777	4.046	0.834	4.12	0.828

*RF, Random Forest. RMSE are in nmol·min^−1^. Colors refer to: linear models (gray) and non-linear models (blue). Models in bold are the top five models for all datasets. Dataset 1 corresponds to the lower part of Entamoeba histolytica glycolysis; Dataset 2 to the peroxide detoxification pathway of Trypanosoma cruzi and Dataset 3 to the industrial-scale penicillin fermentation process of Penicillium chrysogenum*.

For the same reasons stated above, all linear models show poor results in predicting flux starting from enzyme activities, and are therefore not adequate to model the lower part of glycolysis here (Figures 5I–M). Overall and for Dataset 1, the Cubist model has the best generalization capability, with a lower RMSE = 0.154 nmol·min^−1^ and a higher R^2^ = 1 for the test set (Table 3). These results show that the non-linear models, such as random forests, Cubist and XGBoost Linear, are able to indicate the final flux of the pathway by using the predicted data.

### Example 2: The Peroxide Detoxification Pathway of *Trypanosoma cruzi*

#### An *ad hoc* Gray-Box Model Allows Data Augmentation of Enzyme Activities and Flux

**Figure 6:**
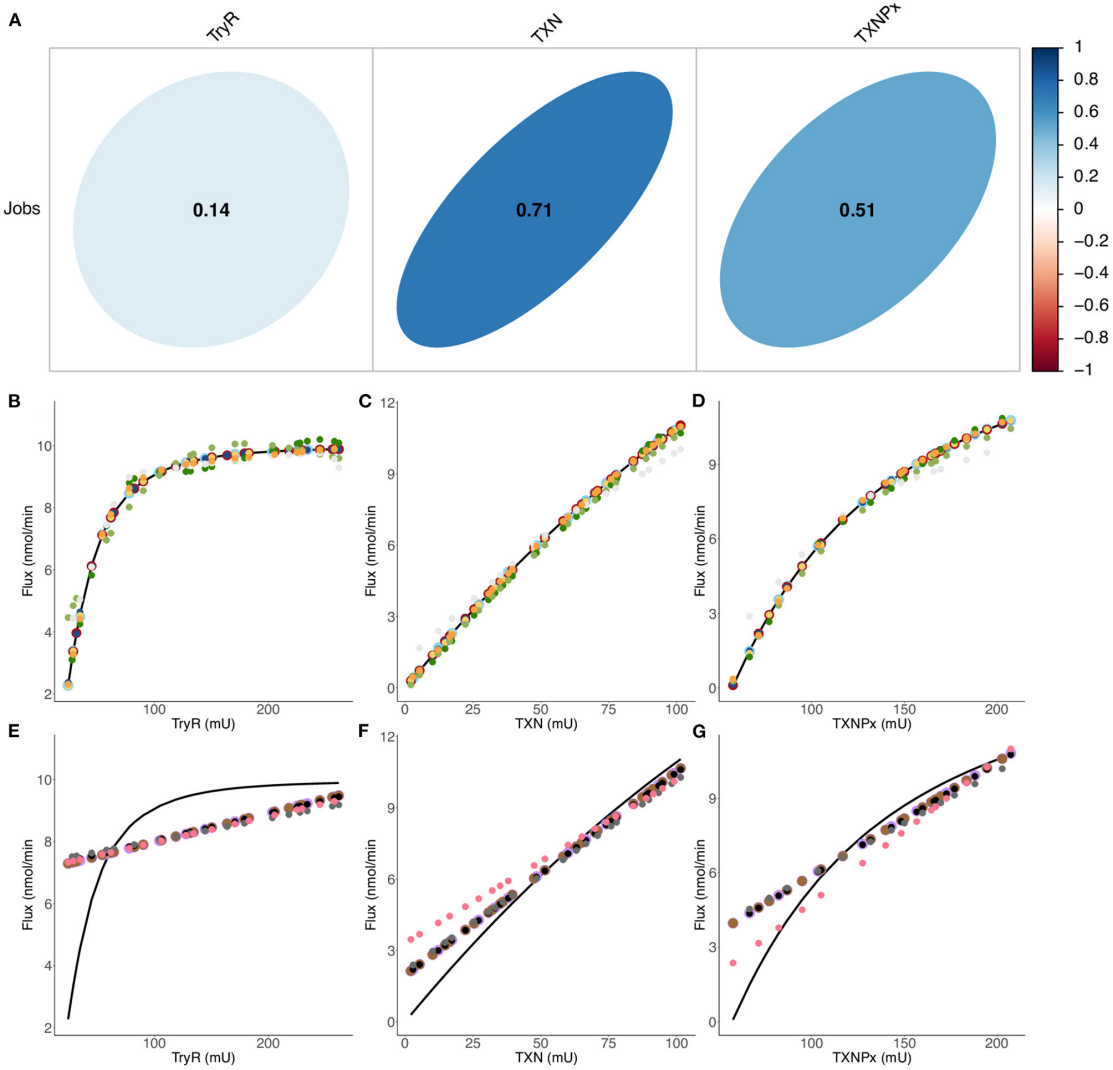
Comparison of final flux predictions by different predictive models. **(A)** Linear correlation between enzyme activities (inputs) and the flux (output) of Dataset 2 (Supplementary Table 8) when all enzyme activities are varied. Correlation coefficients are also calculated when only one enzyme activity is varied: 0.76 (TryR), 0.998 (TXN) and 0.97 (TXNPx). A perfect circle means that there is no linear correlation between the variables, while a straight line means that there is a perfect linear correlation between the variables. **(B–G)** Flux variation as a function of the enzymatic activity of TryR **(B, E)**, TXN **(C, F)** and TXNPx **(D, G)**. Colored circles refer to predicted data from: QRF (dark blue), XGBoost Linear (light blue), Cubist (red), Rborist (yellow), ANN (orange), bagEarth GCV (light gray), SVM Poly (light green), SVM Radial (dark green), Bayesian GLM (purple), Spike-and-slab (brown), Ridge (black), Lasso (dark gray) and PLS (pink). A curve of the fitting experimental data is represented by the black curve. See Table 3 for the statistical measurements of each model.

We look at modeling the second metabolic pathway, which can also be used for drug design purposes. In the gray-box model developed here around this second dataset, the first and third enzymes employ a modified kinetic equation including two different adjustment terms: α =23 and ß = 8 (Table 1). The determination of these parameters is detailed in the Methods section. We obtained a relatively good model of flux prediction (R ^2^≈ 0.67 and RMSE = 4.668 nmol·min^−1^) when enzyme activities are varied (Supplementary Figure 7). However, the model still overestimates the flux when TryR activity is varied and when TXNPx activity is higher than 698.35 mU. The new dataset contains 1,671 enzyme balances evolving around the experimental ones (Dataset 2, see Supplementary Table 8). The predicted final fluxes vary between 0 and 11.46 nmol·min^−1^; the dataset's distribution is shown in Supplementary Figure 8, Supplementary Table 4. It is important to note that we could not go below 16.1 mU and 57.6 mU for TryR and TXNPx activity. The reason is that the gray-box model is not able to predict the flux below these values. Also, an analysis of the correlation between the different variable shows that TXN has the highest correlation coefficient, followed by TXNPx and lastly TryR (Figure 6A). Here, these linear correlation coefficients point out the predominantly non-linear character of this metabolic pathway, when TryR orTXNPx activities is varied. The non-linear aspect of the peroxide detoxification pathway is certainly not to be negligeable, since the coefficient average, when all enzyme activities are varied, is lower than 0.6. These results support those obtained by González-Chávez et al. (2015, 2019) which demonstrate that TXN and TXNPx exert the greatest control on the pathway's flux, while TryR exerts very little control on the flux.

The augmented dataset is now used to test different ML approaches, as described in the following section.

#### Non-linear Machine Learning Methods Are Efficient for Flux Prediction

**Figure 7:**
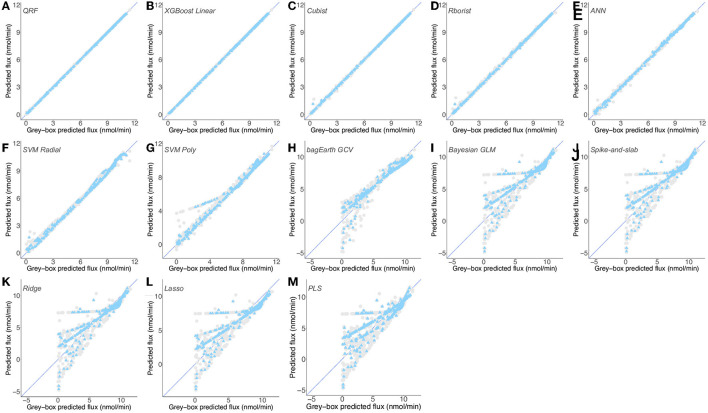
Predictions of gray-box predicted flux by different predictive models for Dataset 2. **(A–M)** Flux from the second dataset (Supplementary Table 8) predicted by the QRF **(A)**, XGBoost Linear **(B)**, Cubist **(C)**, Rborist **(D)**, ANN **(E)**, SVM Radial **(F)**, SVM Poly **(G)**, bagEarth GCV **(H)**, Bayesian GLM **(I)**, Spike-and-slab **(J)**, Ridge **(K)**, Lasso **(L)** and PLS **(M)** models. Gray circles: training set, and blue triangles: test set. See Table 3 for the statistical measurements of each model.

We built different ML models and evaluated their performance. Of the thirteen models built, only five predict well the flux for both training and test sets: the random forest (QRF and Rborist), XGBoost Linear, Cubist and ANN (Figures 6B–D, 7A-E). These models have a cvRMSE range of 0.128-0.186 nmol·min^−1^ and cvR^2^ of 0.996-0.998 for the training set, and RMSE range of 0.022-0.098 nmol·min^−1^ and R^2^ of 0.999-1 for the test set (Table 3). The following three models (SVM Radial, SVM Poly and bagEarth GCV) predict moderately well the flux of peroxide detoxification (Figures 6B–D, 7F–H), with cvRMSE between 0.349 and 0.956 nmol·min^−1^, and cvR^2^ between 0.916 and 0.989 (Table 3). With the test set, their performance is slightly lower, with RMSE between 0.233 and 0.964 nmol·min^−1^ and R^2^ between 0.914 and 0.996 (Table 3).

In contrast, the last five models can hardly predict the flux from enzymatic activities for both training and test sets, particularly for flux below 7.5 nmol·min^−1^ which is within the physiological and experimentally determined value (Figures 6E–G, 7I–M). These models present higher RSME and lower R^2^ values for the training set (cvRMSE range of 1.44-1.581 nmol·min^−1^ and cvR^2^ range of 0.765-0.805) and test set (RMSE between 1.379 and 1.55 nmol·min^−1^ and R^2^ range of 0.777-0.823), confirming their poorer performance not only in terms of learning but also in terms of generalization, in making robust predictions on new data (Table 3). We also observe that models Bayesian GLM, Spike-and-slab and Ridge give comparable results (Table 3 and Figures 7I–K).

These results, together with those in example 1, allow us to confirm that non-linear models are more appropriate to predict the flux of a metabolic pathway than linear ones. Moreover, it should be noted that our gray-box models, built with COPASI, are non-linear models and that the data of Datasets 1 and 2 are mostly obtained with these non-linear kinetic models. To ensure that the preceding results are not influenced by the kinetic model used to generate the data, we use a new raw dataset from experimental records of a bioreactor.

### Example 3: The Industrial-Scale Penicillin Fermentation Process of *Penicillium chrysogenum*

In addition, another type of metabolic pathway we can examine is the production pathways; their modeling would allow the development of an optimized overall process. In fact, another study revealed that ML methods can accelerate the optimization of chemical synthesis (Hein, 2021). As stated before, we do not need to enlarge this dataset, which is composed of records of the various parameters of an industrial-scale penicillin fermentation process. The use of this dataset made of only experimental data will ensure the reliability or not of the ML models for metabolic pathway prediction. It is important to consider that the inputs of our models are no longer the enzymatic activities, but different variables such as: batch time, oil flow, aeration rate, vessel volume and weight, carbon evolution rate and CO_2_ percentage in off-gas. A slight variation of CO_2_ in off-gas is recorded (Supplementary Table 9); this can be explained by the implementation of a system, by the operators, allowing corrective measures to be taken when the CO_2_ level is too high, thus avoiding the detrimental effect of an accumulation of CO_2_ on the growth of *Penicillium chrysogenum* and the production of penicillin. As the percentage of CO_2_ in off-gas is maintained at a certain level, it is not surprising that the carbon evolution rate does not vary much either and presents a low standard deviation (Supplementary Table 5). Also, the output we are interested in is not the pathway flux, but the final concentration of penicillin (Figure 3C). As regards the correlation coefficient between the variables, we note that it is generally high between the parameters and the final penicillin concentration (Table 4); this correlation can be positive (e.g., time) or negative (e.g., oil flow). These correlation coefficients reveal the linear nature of the fermentation process studied in Dataset 3.

**Table 4:** Correlation table between the parameters of the bioreactor and the observed penicillin concentration for Dataset 3.

	***Observed penicillin concentration***
Time	0.92
Oil flow	−0.81
Aeration rate	0.78
Vessel weight	0.79
Carbon evolution rate	0.78
Vessel volume	0.76
CO_2_ in off-gas	0.68

#### Non-linear Machine Learning Methods Predict the Fermentation Process Better Than Linear Methods

**Figure 8:**
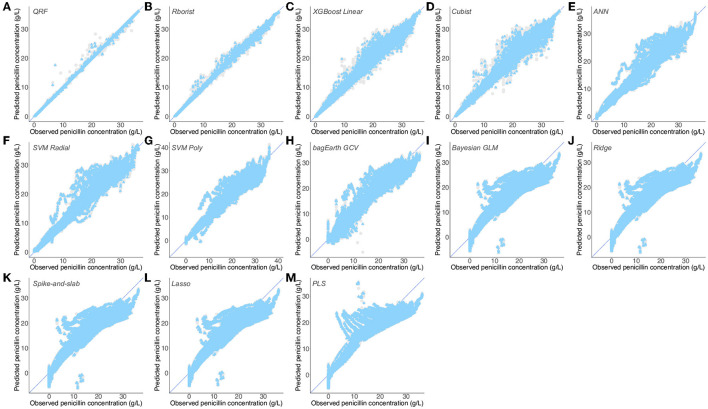
Predictions of observed penicillin concentration by different predictive models. **(A–M)** Flux from the third dataset (Supplementary Table 9) predicted by the QRF **(A)**, Rborist **(B)**, XGBoost Linear **(C)**, Cubist **(D)**, ANN **(E)**, SVM Radial **(F)**, SVM Poly **(G)**, bagEarth GCV **(H)**, Bayesian GLM **(I)**, Ridge **(J)**, Spike-and-slab **(K)**, Lasso **(L)** and PLS **(M)** models. Gray circles: training set, and blue triangles: test set. See Table 3 for the statistical measurements of each model.

The results of penicillin concentration predictions reveal that Random Forest models effectively predict experimental concentrations, with cvRMSE = 0.814/0.877 g·L^−1^ and cvR^2^ = 0.993/0.992 (QRF/Rborist) for the training set and RMSE = 0.134/0.319 g·L^−1^ and R^2^ = 1/0.999 (QRF/Rborist) for the test set (Table 3 and Figures 8A,B).

We can then separate the rest of the models into two groups, based on their performance on the test set. The first one, which predicts the penicillin concentration fairly well, has RMSE between 1.097 and 2.418 g·L^−1^, and R^2^ between 0.941 and 0.988 (Table 3 and Figures 8C–H). By contrast, we found that the predictions of the second group are considerably worse, with many more outliers (Figures 8I–M), and with RSME higher than 3.5 g·L^−1^ and R^2^ lower than 0.9 for the test set (Table 3). As noted in the previous dataset, we also found many models that give the same results, namely: Bayesian GLM, Spike-and-slab, Ridge and Lasso (Table 3 and Figures 8I–L). Here also, Lasso and PLS were the worst in terms of predictions. Interestingly, compared to the preceding results, Dataset 3 gives the best results for linear models (lowest RMSE and highest R^2^ values for the training and test sets); this could be explained by the largely linear nature of the penicillin concentration used with respect to the parameters used. These results support the previous ones and confirm that non-linear models surpass linear models for the prediction of penicillin concentration through the fermentation process.

### Performance Comparison of All Models

**Figure 9:**
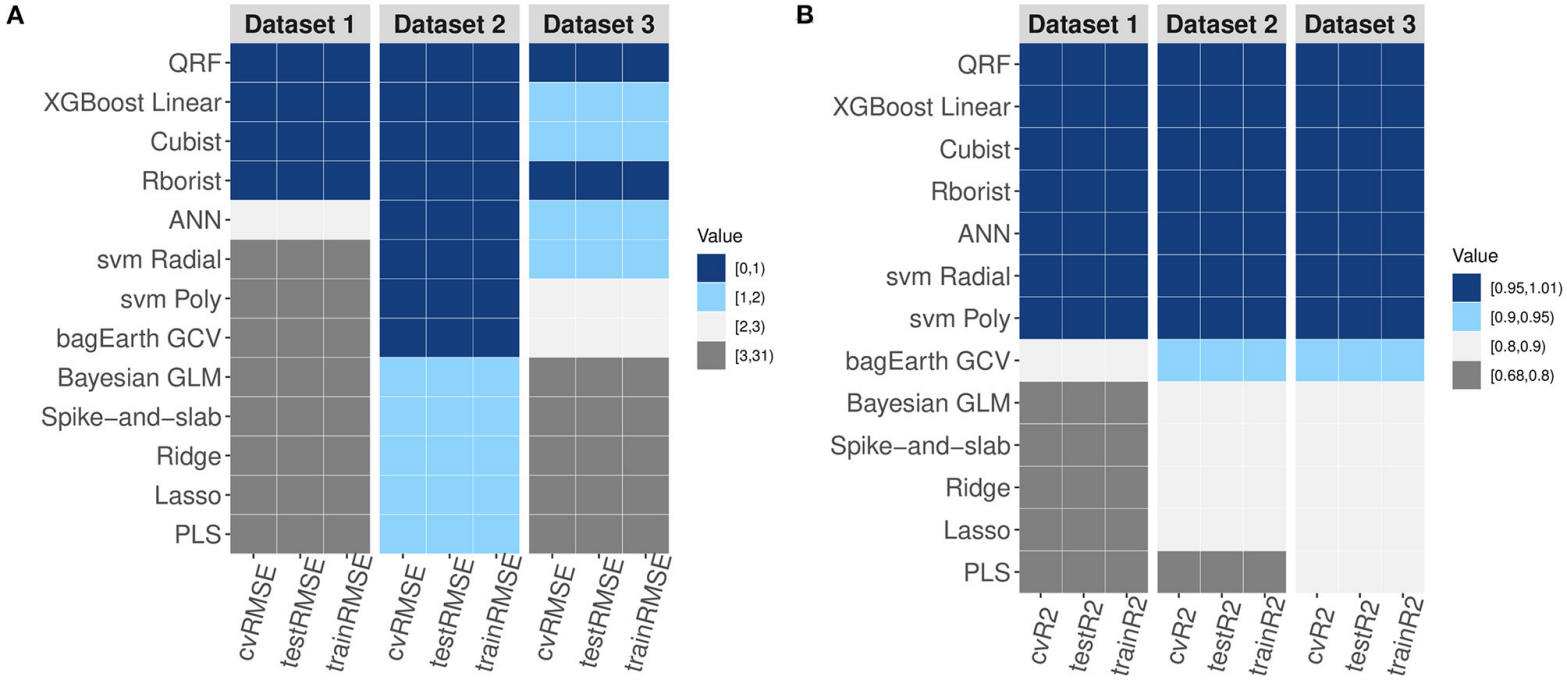
Comparison of the RMSE and R^2^ of the three datasets. **(A,B)** Variation of RMSE **(A)** and R^2^ **(B)** values for the different models and for each dataset.

After showing that non-linear ML methods are more suitable for modeling metabolic pathways, we performed a comparison of the performance of all models. At first glance, the plots further confirm the preceding results and display higher RMSE values and lower R^2^ values for the linear models compared to non-linear models (Figure 9). In addition, regardless of the number and/or type of data, we observe that Spike-and-slab, Ridge, Lasso and Bayesian GLM models give almost the same results (Figure 9 and Table 3). Also, it appears that some non-linear models work less well with large datasets; this is the case for ANN, bagEarth GCV, SVM Poly and SVM Radial (Figure 9). Moreover, it appears that random forest models (QRF and Rborist) are the best suited for metabolic pathway modeling, as they give the best results in term of RMSE and R^2^ whatever dataset was used. Furthermore, we can evaluate the impact of the degree of non-linearity of the pathway on the predictions. Indeed, the pathway that has a high non-linear structure (Dataset 1) gives worse results for linear models than the pathway that presents a less non-linear structure (Dataset 3), which also gives good results with non-linear models (Figure 9A and Table 3). For example, Dataset 1 performs less well with the Ridge model, with RMSE = 29.311 nmol·min^−1^ and R^2^ = 0.716, than Dataset 3, which performs well with the same model, with RMSE = 3.579 nmol·min^−1^ and R^2^ = 0.87.

**Figure 10:**
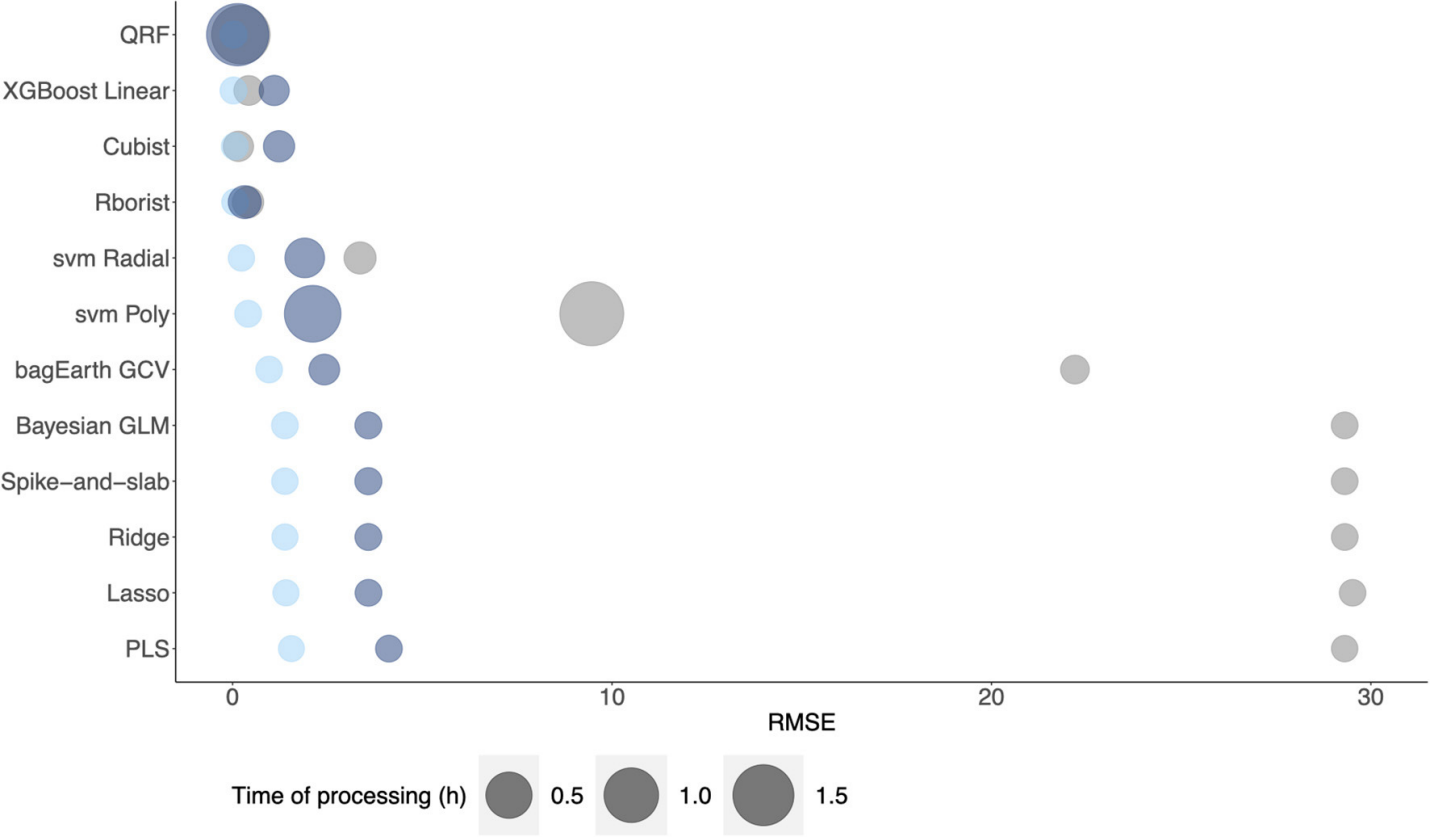
Comparison of model processing time against RMSE of each dataset: Dataset 1 (gray), Dataset 2 (light blue) and Dataset 3 (dark blue) for metabolic flux prediction.

Besides, with a view to applying these methods at an industrial level, we perform a comparison of model error prediction and time of processing among the different datasets (Figure 10). The results confirm the previous findings, where random forest models have the best performance for metabolic pathway flux prediction. We noted that Rborist model presents a better RMSE - time of processing ratio than QRF model. However, even if QRF models have a processing time higher than 1h, we obtain an RMSE gain of about 96 %, when comparing it with PLS model, which could be of considerable significance for the industrial level. In view of the considerable gain of using this method compared to a linear one, non-linear methods could be more beneficial at the industrial level, where a gain of 1% is colossal. Spike-and-slab, Ridge, Lasso and Bayesian GLM models result in comparable performance in terms of RMSE and time of processing. At least, these results show a better RMSE – time of processing ratio for non-linear methods than for linear ones. We did not add the ANN models in the results, as they were not performed using parallelization process compared to the other methods.

Furthermore, we assess the impact of the amount of training data on ML model performance to have a desired level of performance (Supplementary Figure 9). We observe that the results are roughly the same for the datasets when they are predicted with linear models (Supplementary Figures 9B,D,E,H,I), thus the amount of data required to obtain a strong linear model can be higher than 80,000 data, as long as the studied pathway does not have a high degree of non-linearity. When it comes to non-linear models, we find that using a dataset smaller than 40,000 data is sufficient to obtain a good ML model (Supplementary Figures 9A,C,F,G,J–L). Using a dataset higher than 40,000 data leads to non-linear models that are efficient only in case of random forests (QRF and Rborist), Cubist and XGboost Linear methods, for which RMSE is low. We could also consider making an ablation of our datasets to examine the impact of amount of training set data on the ML model performance.

## Discussion

### Comparison and Applicability of Knowledge-Based and Data-Driven Approaches

The first objective of this study is to determine what sort of data-driven model could better simulate the biological pathways studied. By using different datasets, we build several models with the enzyme balances or parameters collected from a bioreactor and reveal that Random Forests (QRF and Rborist), Cubist and XGBoost Linear are three good methods to predict the final flux or concentration of a final product. This works is part of a larger study about the applicability of either a knowledge-based or a data-driven approach. Indeed, in other fields such as fault detection and diagnosis, a comparison of these two methods demonstrates that they both have comparable performance and can be used (Alzghoul et al., 2014; Yang and Rizzoni, 2016). In biological system modeling, as is the case here, we demonstrated that in instances where little knowledge is available and difficult to obtain on a large scale basis (e.g., kinetic parameters k_cat_ and K_m_ of an enzyme, pathway fluxes), or when complex feedback regulation mechanisms take place, a data-driven method can be a good alternative for modeling a metabolic pathway, as many authors have shown before (Ramachandran et al., 2011; Hou et al., 2016). By comparison, the knowledge-based method can be laborious and long, due to data mining from the literature or wet laboratory experiments, whereas there is ease and speed of building models with the data-driven method (Kadarmideen, 2016).

Another criterion that we considered was the degree of non-linearity of the pathway. As mentioned above, it is generally admit that metabolic systems have an inherent non-linear behavior (Koza et al., 2001; Song and Ramkrishna, 2013; Yasemi and Jolicoeur, 2021). However, there is no formal demonstration of the non-linear structure of metabolic pathways. According to Song and Ramkrishna, this non-linear behavior would be due to: (i) the non-linearity of the chemical reactions forming the pathway and (ii) the regulatory processes that added non-linearity to the system (Song and Ramkrishna, 2013). Also, it is expected that pathway fluxes are non-linear, because they are controlled by enzymes and the activities of metabolic enzymes are saturable by their ligands. Besides, when the fluxes are measured in intact cells, they give a non-linear behavior and flux variation appears as hyperbolic or even sigmoidal. If the measured fluxes appear linear, it might be because the saturation point is not reached. Furthermore, according to the Metabolic Control Analysis, the fluxes are hyperbolic or non-linear because always exist one or two flux-controlling steps which utlimately determine the pathway flux (Fell, 1992). The determination of linear correlation coefficients of the different variables of the datasets gives us insights into the degree of non-linearity of the studied metabolic pathways and provides a method to evaluate the non-linearity of metabolic pathways. We found that all metabolic pathways studied here have a notable non-linear structure, with Dataset 1 having the highest degree of non-linearity, then Dataset 2 and lastly Dataset 3. These results generally comfort the main hypothesis that metabolic pathways are predominantly non-linear. The determination of the degree of non-linearity is therefore important for selecting and applying of a ML technique when modeling a metabolic pathway.

Moreover, the suitability of using either method relies on the quantity and quality of the knowledge or the data. Here, to illustrate this point, we simulate two datasets: the first one consisting of an exploration of the experimental data (2,000 data) and the second one composed of enzyme activities from 0 to 1,000 mU (68,950 data). The largest one gives better predictions for the three best models (Random forests, Cubist and XGBoost Linear) than the other dataset, and shows us the importance of having a large dataset before using machine learning methods. In fact, the size of the training set has been shown to be a major driving factor of prediction accuracy (Somarathna et al., 2017). However, we used two datasets made up of a mix of experimental and predicted data to build the models, and even if predicted from a good quality model, they remain mostly predicted data and are not comparable to a fully experimental dataset, which is also difficult to obtain. Thus, it would be worth considering methods using only experimental data, when sufficient data are available to build the models. Interestingly, a data-driven approach is often used to discover biological pathways or unravel pathways that are not well understood. Thus, combined with the knowledge-based approach, this can quickly make clear the complexity of biological systems modeling. Another possibility would be to test ML models on experimental data from *E. coli* or yeast, which can present a larger degree of non-linearity and are easily found in the literature. This issue will be addressed in our next study.

Surprisingly, model performance was weaker for the largest dataset from the bioreactor records than for the smaller datasets. The reason for this result may lie in the choice of input variables. Several studies have highlighted the need for variable selection in order to have better predictions (Camacho et al., 2018; Awan et al., 2019; Genuer et al.). Indeed, variable selection allows the use of the most informative variables to predict the output variable(s) and reduce the time of computing. Unlike the knowledge-based model, a diversity of variables for data-based models does not always mean better performance. This is one of the limitations of our study, since only one combination of input variables was tested during the work. It would be interesting for a future study to compare, for the same dataset, models using different sets of input variables, and to analyze their impact on model effectiveness.

### Interpretability of Machine-Learning Approaches

Another major issue facing users of machine learning approaches is the interpretability of these models. Even if, at this time, we do not have a common general definition of this term, many researchers, such as Schmidt et al. (2019), define a model's interpretability based on two aspects: (a) intrinsic interpretability (or transparency): the ability to understand the inner mechanism of the model in the context of the study (e.g., identification of variables most involved in the predictions), and (b) *post hoc* interpretability: the ability to extract new information from the model or provide new insights into the relationships discovered during the process (e.g., the effect of a variable on another one) (Murdoch et al., 2019; Schmidt et al., 2019; Pintelas et al., 2020). Although some ML methods, such as decision trees or linear regression models, are easily interpretable; this is not the case for most of the models developed here (e.g., XGBoost Linear, bagging MARS, ANN). Nevertheless, using the variables that are directly related to the variable to be predicted, as we do here, allows us to gain some understanding of how the model works and the types of relationships that are revealed, enabling us to rely on the models. Furthermore, while we identified Random forests as one of the best methods for predicting final flux or product concentration, Pintelas et al. (2020) classifies it as a model that is hard to interpret. Therefore, it would be interesting to compute variable importance or to apply different techniques to explain the model in order to increase its interpretability (Zhou et al., 2019; Azodi et al., 2020). Besides, knowing that models based on decision trees are among the simplest to interpret, we support the idea of Schmidt et al. that RF models are more accessible than others from an interpretability point of view (Schmidt et al., 2019). An alternate solution would be to develop simpler models, but this would certainly reduce their overall performance.

Moreover, one of the key factors in the interpretability of the models is linked to the equations used. In fact, compared to knowledge-based models that use well-defined equations with a biological significance, ML models are governed by other equations, which sometimes are “outside our understanding” as Schmidt et al. (2019) observed in their study of the applications of ML in solid-state materials science. This raises a real problem of confidence in the prediction results obtained with such methods. As these authors point out, the fact that these models were not based on physical principles in their studies, or on biological principles in ours, could result in wrong predictions in completely unexpected cases, while providing great results overall. And in the present case where the models are used in the context of biomarker identification or optimization of an industrial bioreactor, we cannot risk obtaining such results from our models in these specific situations. Far from hindering us in the use of ML models, awareness of these problems allows us to formulate several recommendations for future research. These include the combination of interpretable models, e.g., knowledge-based kinetic models with ML models, e.g., random forests models; the prediction of a new set of experimental data with unexpected values. In this latter instance, this would require experimentally testing a range of “extreme” data that would be found in the parasites studied, or recording the bioreactor data even during failures of the penicillin production.

### Strengths and Weaknesses of the Modeling Methods

After analyzing the interpretability of the different modeling methods, it is worthwhile to note some advantages and disadvantages of their use in flux and concentration prediction. One of the best methods in our case is the random forest (QRF and Rborist). Many studies report the use of random forest in the biological field for the prediction of: protein interaction (Qi et al., 2006), drug response based on protein markers (Ma et al., 2006) and *in vitro* drug sensitivity (Riddick et al., 2011). Also, Riddick et al. used SVM and random forest to predict the flux of N_2_O emissions, and found that random forest achieves the best performances among the built models (Villa-Vialaneix et al., 2010). They highlighted that these models offered the advantage of having a low computational cost, compared to the SVM method. However, in our case, we notice that random forest is the least accurate predictability model compared to SVM methods, with the highest computation time for almost all datasets. Moreover, among the random forest packages developed on R, Rborist is quite a recent implementation, designed for multicore hardware, which minimizes data movement within memory to increase the performance and decrease the processing time (Wright and Ziegler, 2017). Surprisingly, here, Rborist package is the one that has the longest time of computation and is more efficient on big datasets compared to other methods. It would be of interest to create variant models combining the random forest method and other methods, as in previous studies (Chen et al., 2018; Zampieri et al., 2019). An existing variant of random forests is the quantile regression forest (QRF) method, which has the capability of establishing prediction intervals that cover uncertainties, useful in the prediction of possible new data (Meinshausen, 2006). Francke et al. demonstrated in their work that this method had the advantage of calculating uncertainties associated with the predicted sediment yields, through the calculation of confidence intervals (Francke et al., 2008). But they also stated that the model predictions will always be within the range of observations, which prevents implausible values but inhibits prediction outside the range of values learned from the training set. We saw here that, overall, QRF models have a good generalization capability; additional prediction of new experimental data, with data separated by a larger stepsize (>25), would be beneficial to confirm or invalidate this capability. This could be useful for the study of metabolic pathways in extremotolerant organisms.

This leads us to note one of the advantages not only of the QRF method but also of other ensemble learning methods, such as XGBoost Linear: prediction from high-dimensional data. Indeed, these models are among the best we have, with any starting dataset we have, from the simplest to the most complex with several types of variables. Remarkably, compared to other models, XGBoost Linear is better ranked for small datasets. This is confirmed by the work of Yang et al. (2010) which propose that ensemble methods have the advantage of reducing the potential for overfitting in small sample size problem. Another strength of XGBoost Linear compared to its peers is the combination of high accuracy and a short time of processing. However, despite the great accuracy of these models, they are often more complex and less interpretable, and present a higher computational intensity.

Moreover, Cubist, a model based on modified regression tree theory, has the advantage of analyzing big data with high speed (Xu et al., 2018). This was confirmed by our results, which show that Cubist is one of our best models (e.g., for Dataset 1, Cubist: 2.49 min and QRF: 1.76 hr). However, we noted that the performance was better for the small datasets than for the bigger one. Another advantage that Das et al. noticed is the fact that the Cubist model is easy to interpret and is a suitable method for beginners (Zhou et al., 2019; Das et al., 2020).

The PLS method turned out not to be appropriate here to model these pathways and predict the final flux starting from enzyme activities, or the final product concentration starting from parameters of a bioreactor. This may be due to the inherent limitation of the PLS method to capture the non-linearities of the metabolic pathways. However, it performs better when we have a smaller dataset, as it has also been noted in a previous study on gluconeogenic flux prediction (Antoniewicz et al., 2006). But these results contradict those obtained with the PLS model for the prediction of limonene and isopentenol synthesis. In fact, in this work, results showed that the model performed well when the dataset was larger (lower RMSE, better predictions) (Costello and Martin, 2018). Also, one big advantage of the PLS technique remains that it has the shortest calculation time for modeling.

It is relevant to observe that the model implementation will differ depending on varying levels of data. In fact, a ML model will be more difficult to implement, if the available data is limited. In this case, a significant additional time is required. Among the various studied models, the difficulty to implement the model could also be based on the higher number of parameters to adjust during the training time.

Our findings generally support the idea that non-linear models are more suitable than linear ones for modeling metabolic pathways. Furthermore, it would be interesting to apply these ML models on genome-scale metabolic networks for which the literature abounds in data. Recently, hybrid models coupling a genome-scale model and ML model have been found to be effective for different purposes such as the prediction of individual amino acid concentration in culture medium (Schinn et al., 2021) and identification of prognostic metabolic biomarkers in cancer studies (Lewis and Kemp, 2021). One of the benefits that ML models could bring is the integration of multi-omics data as genomic, transcriptomic, metabolomic and proteomic data. This topic will be addressed in an upcoming study.

As far as we know, genome-scale models have a predominant place in the field of metabolic networks for the identification of key-molecules in the metabolism. This study allows us to consider the machine learning methods as performant models to predict metabolic pathways. Indeed, their ability to take over large datasets makes them applicable techniques to efficiently predict larger metabolic pathways (e.g., *E. coli*). While flux balance analysis (FBA) based methods, as used in the genome-scale models, need information about the pathway in a given condition as they are hypothesis-driven, machine learning models could predict the metabolic pathways without needing to clearly understand the underlying biological mechanisms of the pathways. Also, constraint-based model (e.g., FBA) are not able to predict metabolite concentrations, while the machine learning methods can consider these predictions. We can thus envisage a hybrid method using both machine learning and FBA methods for metabolic pathway modeling (Zampieri et al., 2019).

### Decision-Making Support for Pathway Modeling

**Figure 11:**
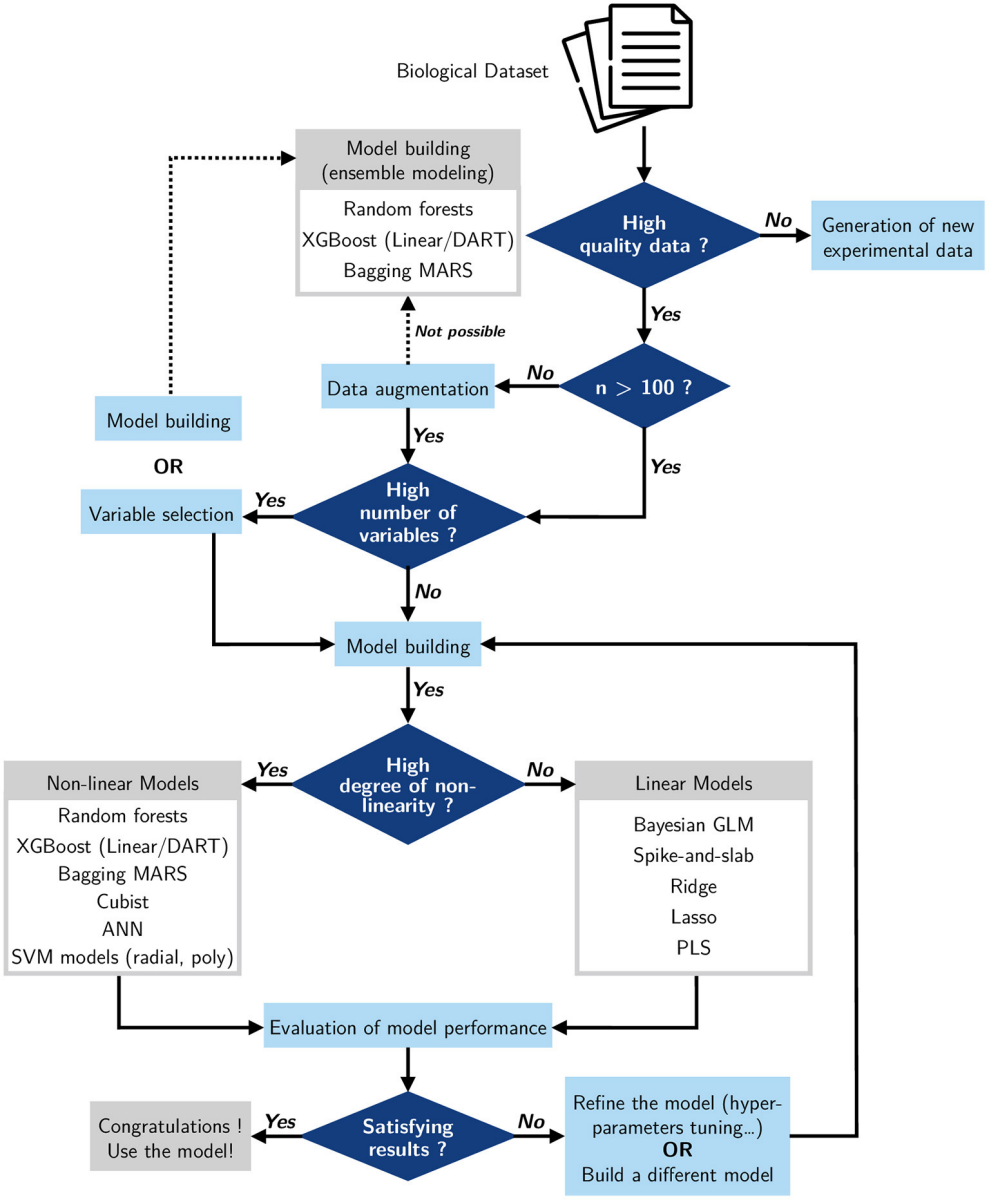
Decision-making support for the construction of metabolic pathway models using machine learning methods.

Given the many different methods that exist and continue to emerge, one can struggle with the choice of a model to build from a dataset. Faced with this decision, we can choose to build simple models or to use models being used in the same field of study and giving good results (Camacho et al., 2018; Cifuentes et al., 2020). In view of this, it would be useful to review and define some basic rules for building a decision-making support for future studies on modeling metabolic pathways. The first feature to consider is the quality of the biological dataset (Figure 11). Do we have an initial dataset of good quality? Data quality can highly impact the model predictions. If the model is not of good quality, it would be better to build a new dataset and generate good quality experimental data. When the dataset is of good quality but small in size, it is useful to do data augmentation, as we did in this study; if this is not possible, we can use an ensemble model to build the metabolic pathway, since such models can deal with small datasets. Another useful criterion we can investigate is the number of variables. If the dataset presents a high number of variables, we can consider doing variable selection before building the model, or we have the option of building the model by using ensemble modeling that gives good accuracy with several input variables. Also, one key factor is the non-linearity of the studied metabolic pathway; do we have a non-linear or a linear process? If our pathway is linear, we can design a battery of linear models which will give a high performance. But if our study involves a pathway that is non-linear, then it is preferable to use a non-linear model. After building our model, an evaluation of its accuracy is necessary to validate it. In case the performance of the model is not suitable, we can plan to refine it, for example by tuning the hyperparameters (Chicco, 2017), or simply to replace it and build a new one.

Non-linear machine learning methods enable us to model metabolic pathways by identifying key-molecules, which are important for the drug-design process, improving disease diagnosis (cancer, viral/parasitic/bacterial infections, neurodegenerative diseases) by highlighting the differences between healthy and pathological situations, or even optimizing industrial production processes.

## Data Availability Statement

The data that support the findings of this study are available from the corresponding author upon reasonable request. The custom codes for the data analysis used in this study are available from the corresponding author in the Github repository: https://github.com/ophelielt/Lo-Thong_et_al._Non-linearity_of_metabolic_pathways_influences_the_choice_of_ML.git.

## Author Contributions

FC, CD, and PC designed the method. OL-T-V, CD, PC, BG-P, XFC, ES, and FC participated in the design of the study and performed the analysis. OL-T-V and XFC wrote algorithms. OL-T-V, CD, PC, XFC, ES, and FC wrote and corrected the manuscript. All authors read and approved the final version of the manuscript.

## Funding

OL-T-V was supported by a PhD grant from the Region Reunion and European Union (FEDER) under European Operational Program FEDER REUNION – 2014/2020 file number 20171389, tiers 216275. Peaccel was supported through a research program partially co-funded by the European Union (UE) and Region Reunion (FEDER). Research at ES laboratory is supported by CONACyT-Mexico grant 282663. The funding agencies had no influence on the research process. XFC was supported by the UKRI CDT in AI for Healthcare http://ai4health.io (Grant No. P/S023283/1).

## Conflict of Interest

The authors declare that the research was conducted in the absence of any commercial or financial relationships that could be construed as a potential conflict of interest.

## Publisher's Note

All claims expressed in this article are solely those of the authors and do not necessarily represent those of their affiliated organizations, or those of the publisher, the editors and the reviewers. Any product that may be evaluated in this article, or claim that may be made by its manufacturer, is not guaranteed or endorsed by the publisher.
