# Supplementary material for: Non-linearity of Metabolic Pathways Critically Influences the Choice of Machine Learning Model
Source: Front Artif Intell. 2022 Jun 10;5:744755. doi: 10.3389/frai.2022.744755 (PMC9226554; doi:10.3389/frai.2022.744755)
Supplement: Supplementary file 1 [file Data_Sheet_1.pdf]

Supplementary Material

Manuscript title: ***Non-linearity of metabolic pathways critically influences the choice of machine learning model.***

1 Supplementary Figures

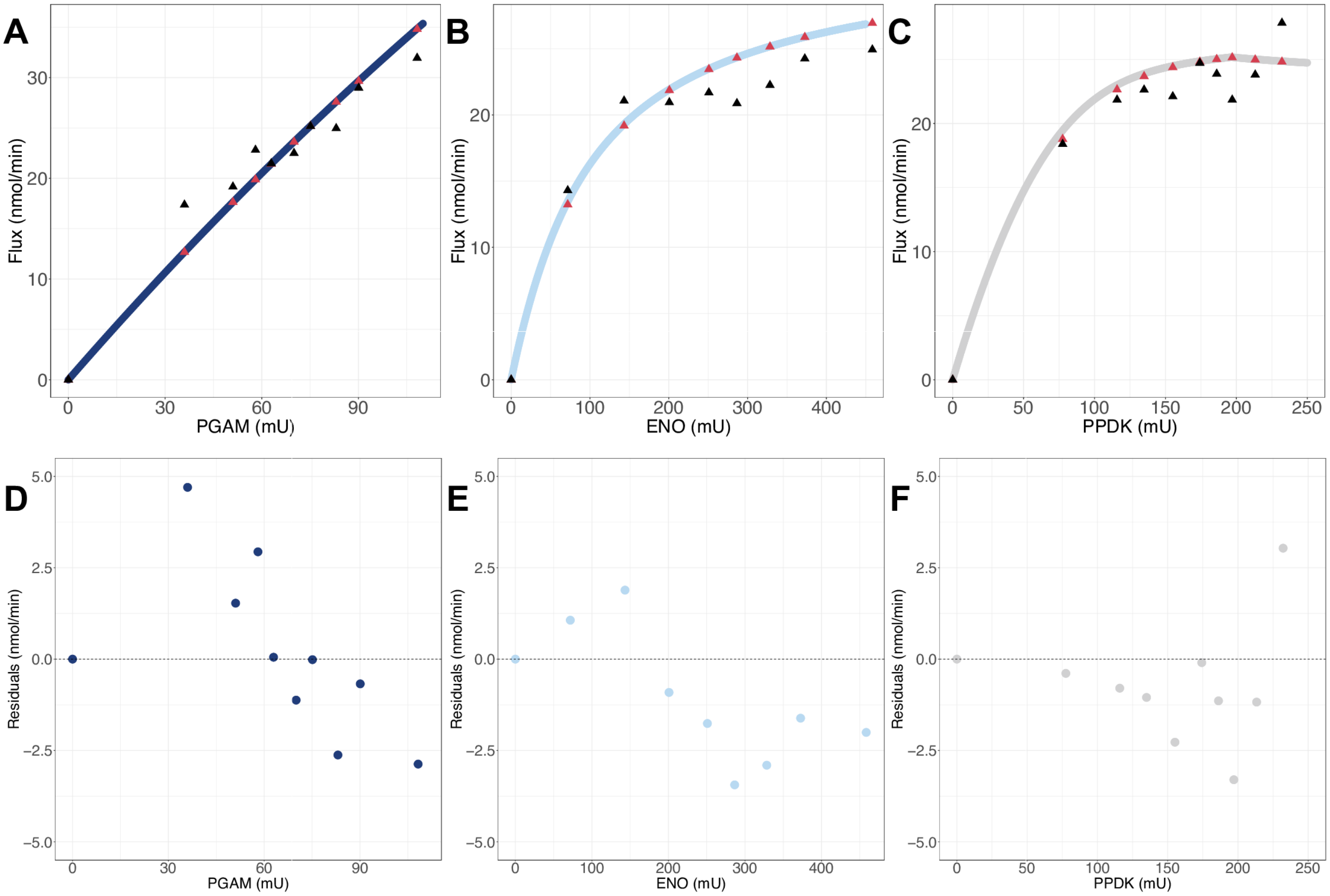

Supplementary Figure 1. Flux predictions with the grey-box model. **(A, B, C)** Flux variation according to PGAM **(A)**, ENO **(B)** or PPDK **(C)** activity. Data are taken from **Supplementary Table 6**. Experimental fluxes are in black triangles, their corresponding predicted flux values are in red triangles and the predicted fluxes are in dark blue for PGAM, light blue for ENO and grey for PPDK. **(D, E, F)**. Residuals for the experimental values versus PGAM **(D)**, ENO **(E)** or PPDK **(F)**.

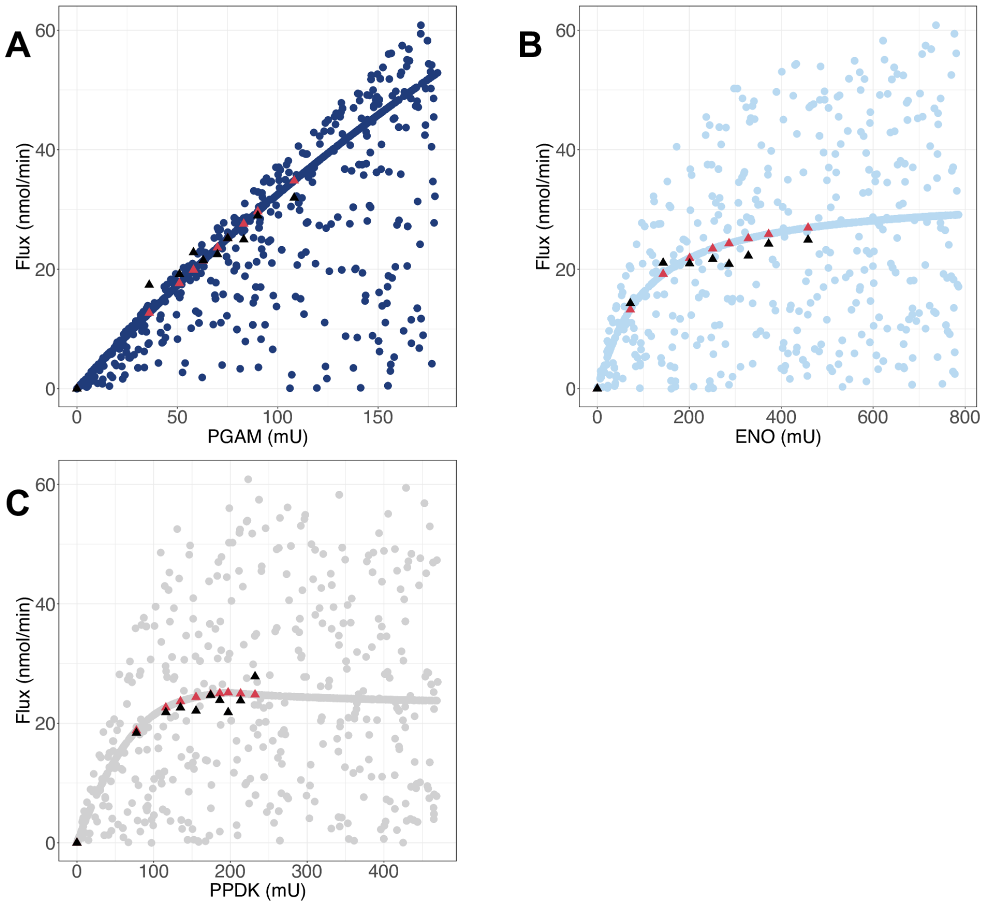

Supplementary Figure 2. Flux predicted for the first dataset (2,000 data). **(A, B, C)** Flux variation according to PGAM **(A)**, ENO **(B)** or PPDK **(C)** activity. Predicted flux from the experimental dataset are in red diamonds and the corresponding experimental flux values are in black diamonds.

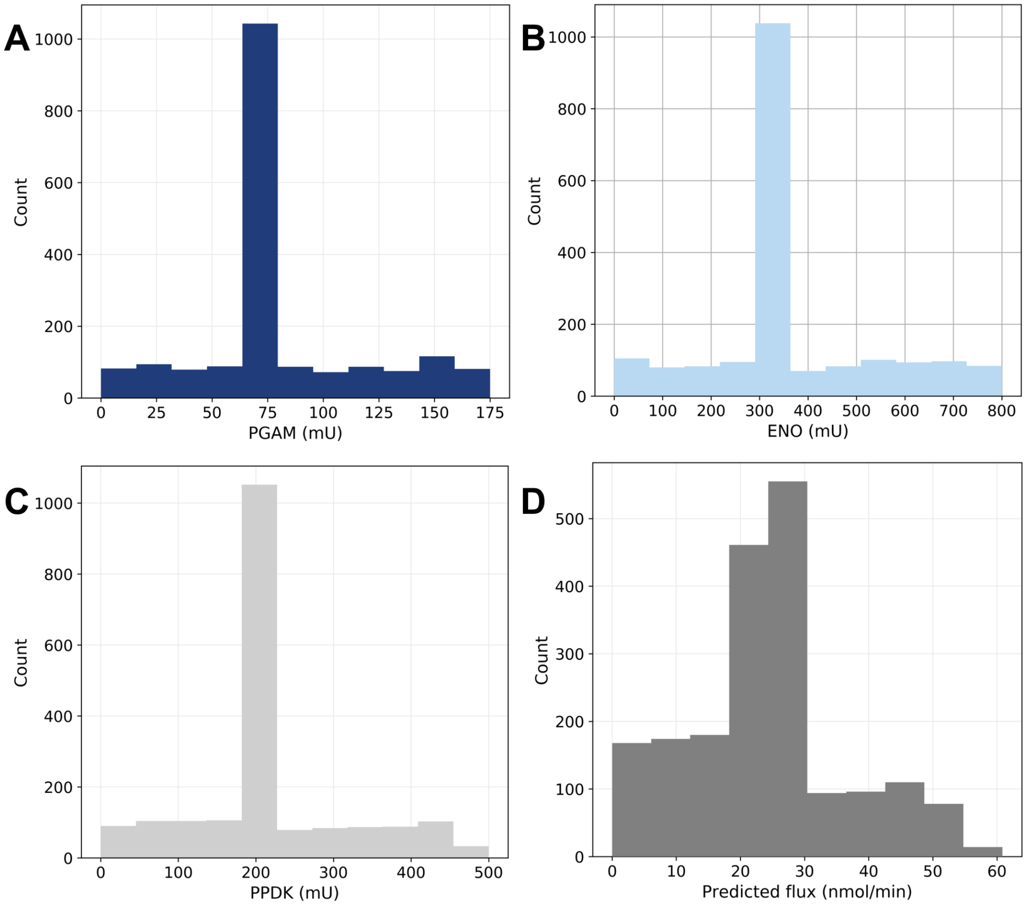

Supplementary Figure 3. Histogram of the first dataset distribution. **(A, B, C)** Variation of PGAM **(A)**, ENO **(B)** or PPDK **(C)** activity. **(D)** Variation of the final flux predicted by the grey-box model.

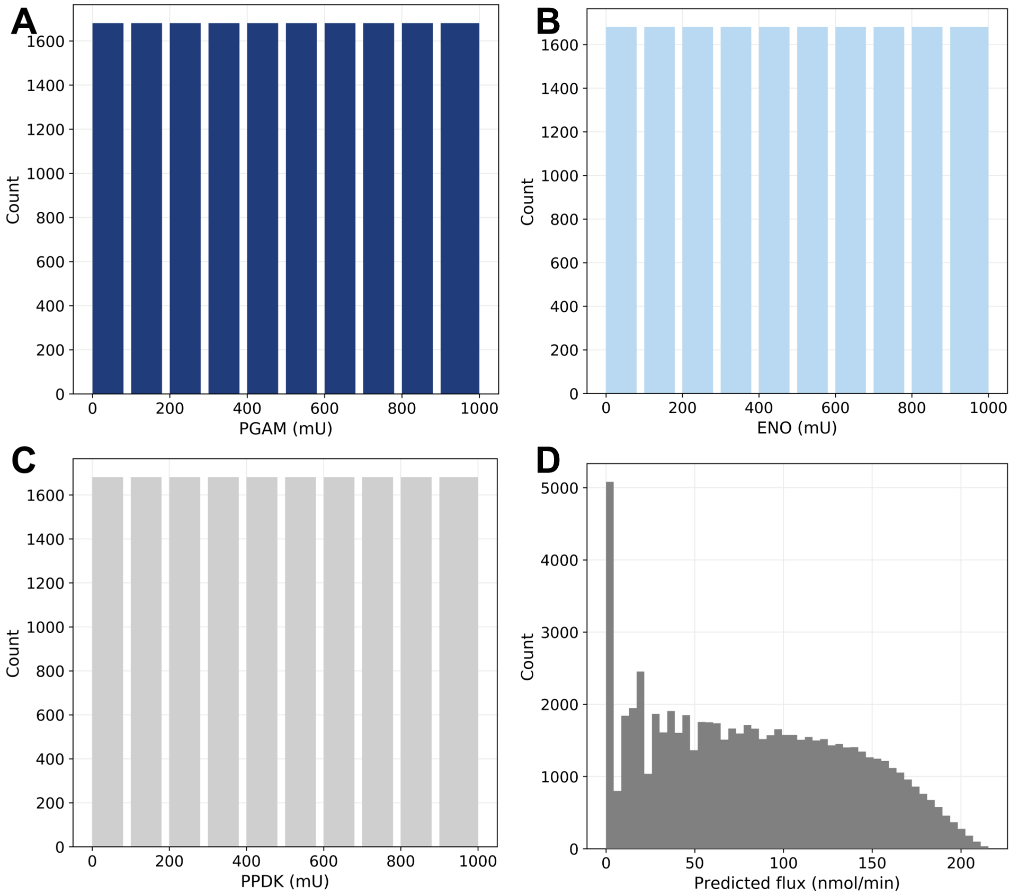

Supplementary Figure 4. Histogram of the distribution of Dataset 1 (68,950 data). **(A, B, C)** Variation of PGAM **(A)**, ENO **(B)** or PPDK **(C)** activity. **(D)** Variation of the final flux predicted by the grey-box model. The physiological *in vivo* fluxes are around 50 nmol·min^{-1}.

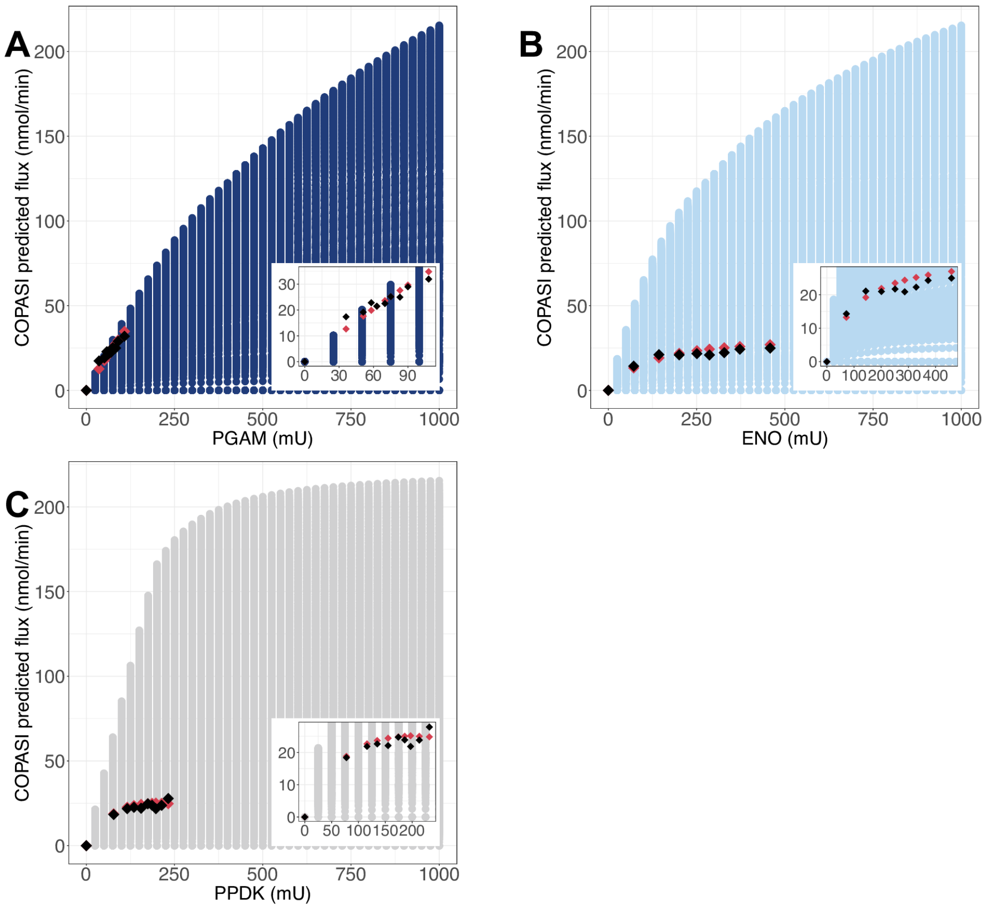

Supplementary Figure 5. Flux predicted for Dataset 1 (**Supplementary Table 7**). **(A, B, C)** Flux variation according to PGAM **(A)**, ENO **(B)** or PPDK **(C)** activity. Predicted flux from the experimental dataset are in red diamonds and the corresponding experimental flux values are in black diamonds.

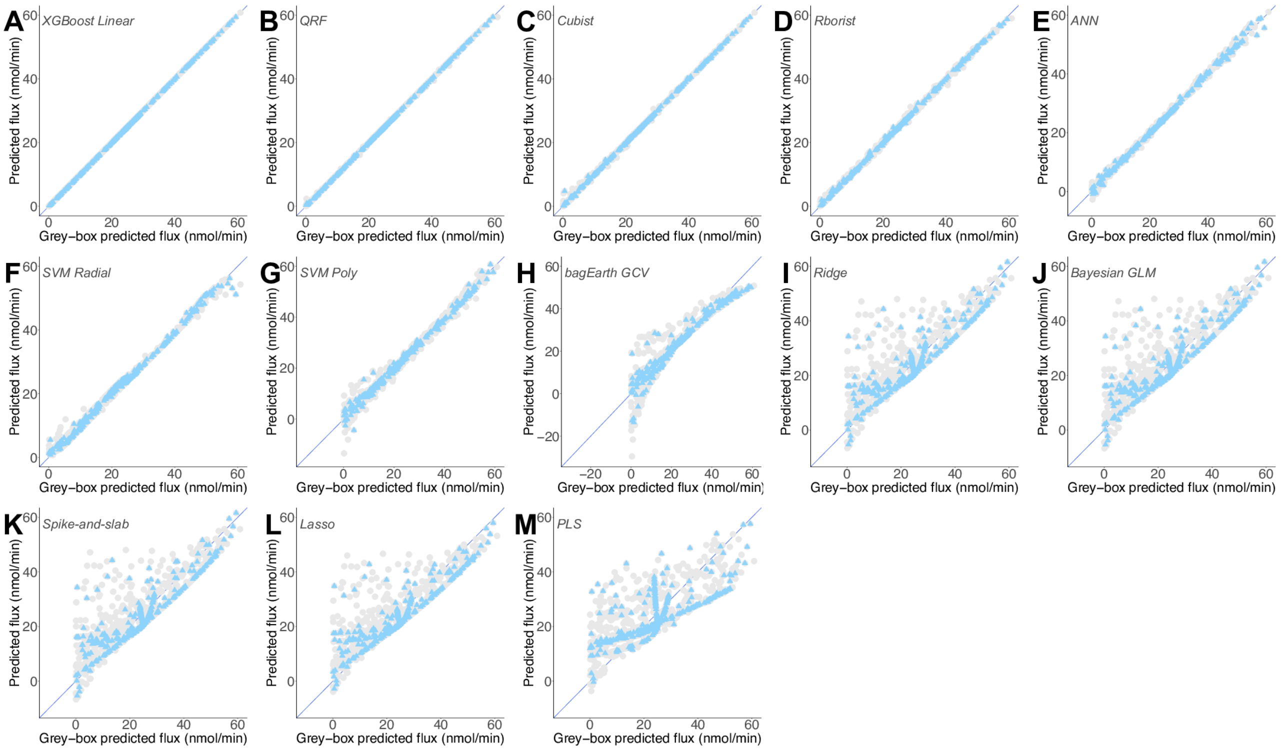

Supplementary Figure 6. Predictions of mix of experimental and grey-box predicted flux by different predictive models. **(A-M)** Flux from **Supplementary Table 6** predicted by the XGBoost Linear **(A)**, QRF **(B)**, Cubist **(C)**, Rborist **(D)**, ANN **(E)**, SVM Radial **(F)**, SVM Poly **(G)**, bagEarth GCV **(H)**, Ridge **(I)**, Bayesian GLM **(J)**, Spike-and-slab **(K)**, Lasso **(L)** and PLS **(M)** models. Grey circles: training set, and blue triangles: test set. See Table S4 for the statistical measurements of each model.

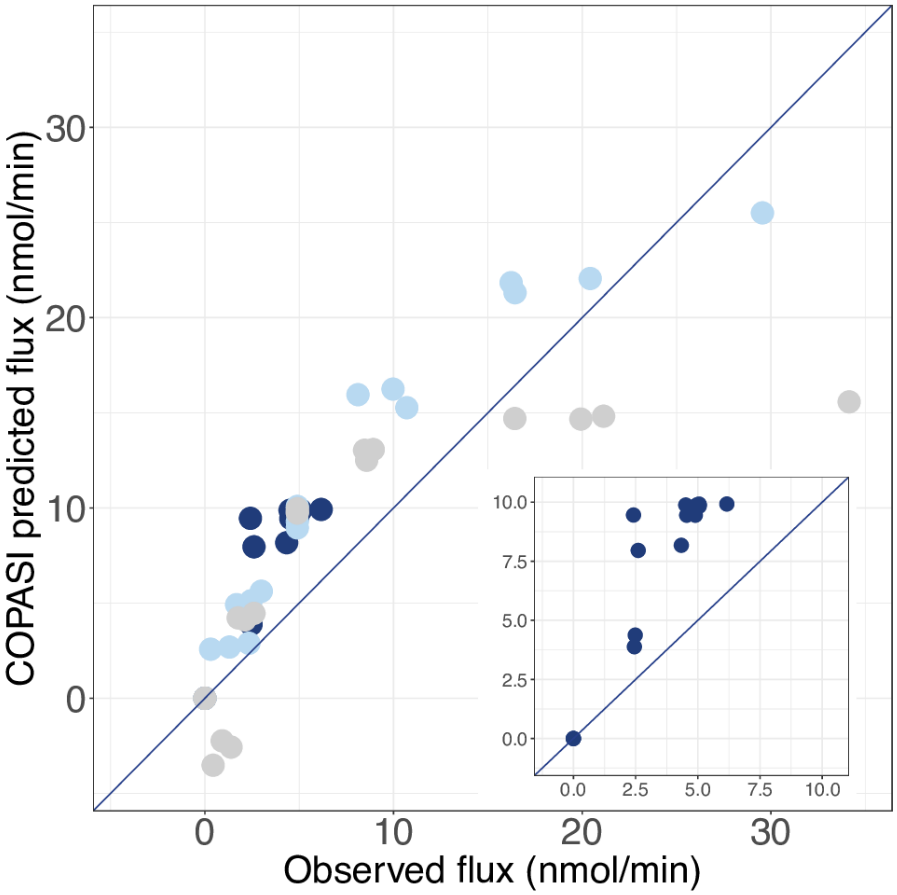

Supplementary Figure 7. Flux predictions by the grey-box model. Circle colors refer to the various levels of enzyme activity: TryR (dark blue), TXN (light blue) or TXNPx (grey). Inset: flux predicted when TryR activity is varied.

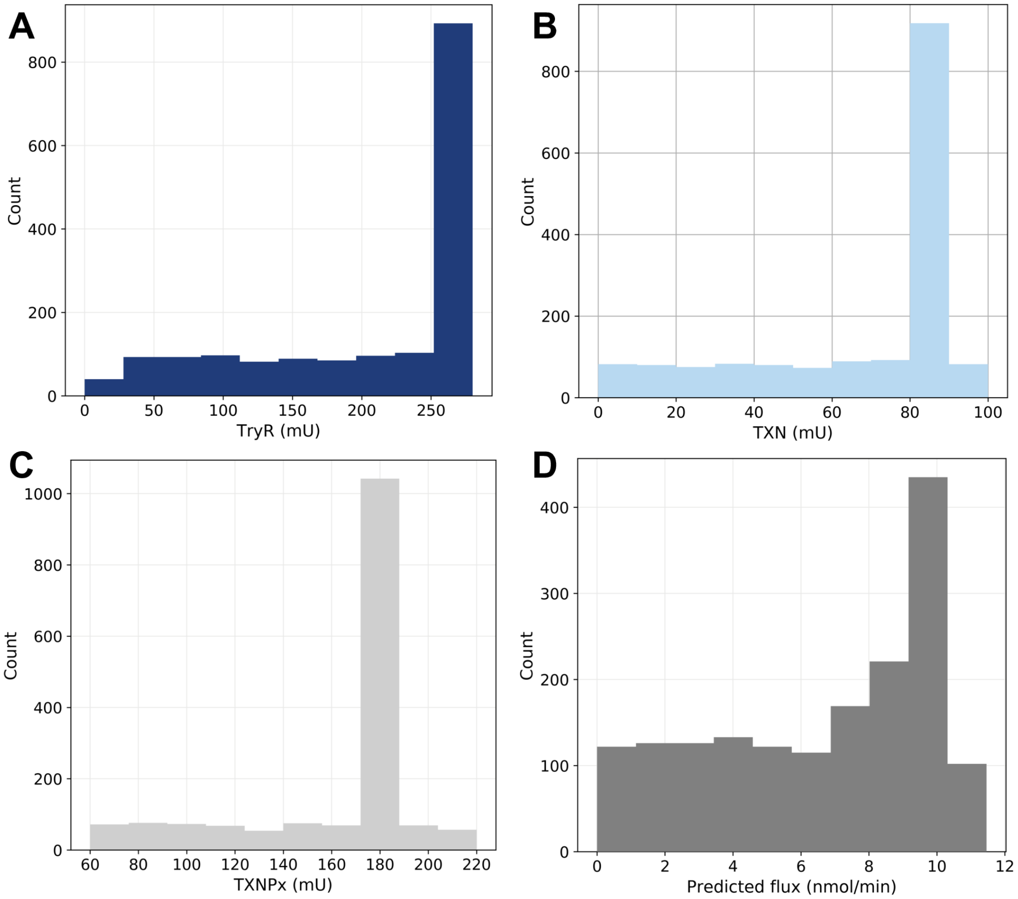

Supplementary Figure 8. Histogram of the distribution of Dataset 2. **(A, B, C)** Variation of TryR **(A)**, TXN **(B)** or TXNPx **(C)** activity. **(D)** Variation of the final flux predicted by the grey-box model.

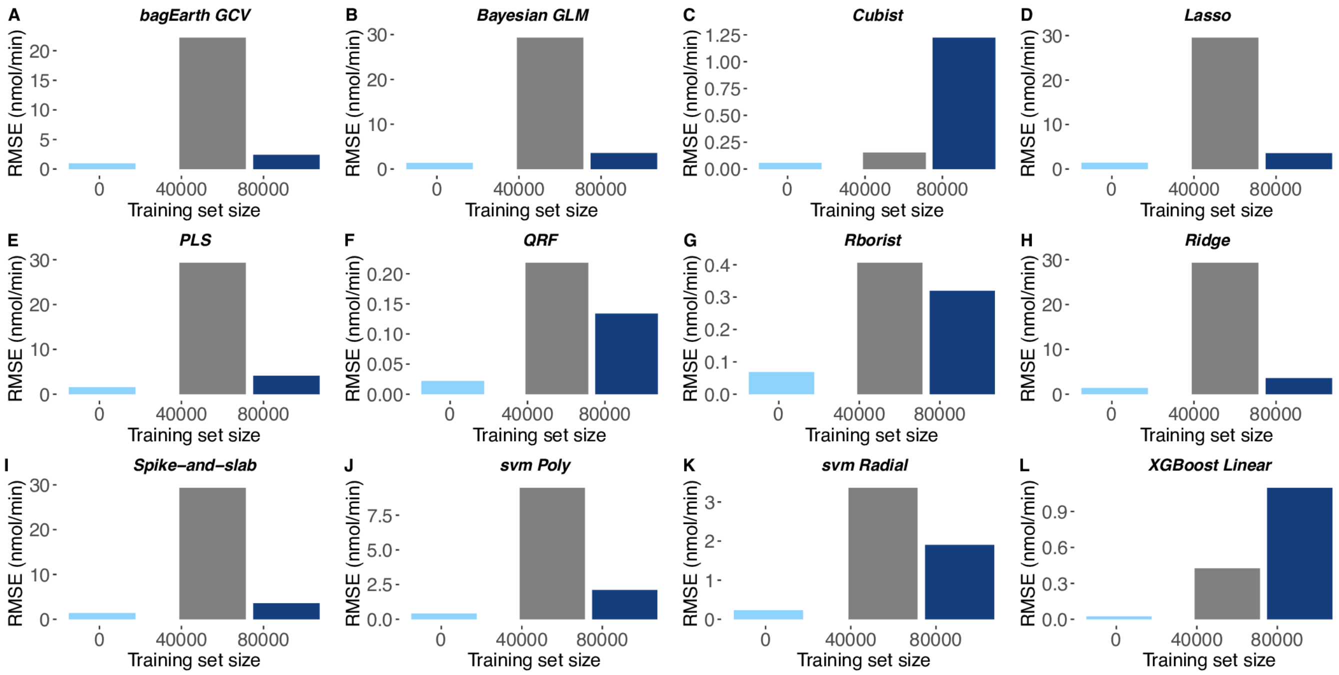

Supplementary Figure 9. Impact of the amount of data on the performance of machine learning models **(A-L)**: bagEarth GCV **(A)**, Bayesian GLM **(B)**, Cubist **(C)**, Lasso **(D)**, PLS **(E)**, QRF **(F)**, Rborist **(G)**, Ridge **(H)**, Spike-and-slab **(I)**, svm Poly **(J)**, svm Radial **(K)**, XGBoost Linear **(L)**. Bar colors refer to the datasets: Dataset 1 (grey), Dataset 2 (light blue) and Dataset 3 (dark blue).

2 Supplementary Tables

Supplementary Table 1. Kinetic equations used in the grey-box model of the lower part of glycolysis (Lo-Thong et al., 2020).

K_m is the Michaelis constant of the enzyme; K_i is the inhibitor constant; V_f and V_r are maximum rates of the forward and reverse reactions; K_{eq} is the equilibrium constant of the reaction.
[a] A, B and C and K_{mA}, K_{mB} and K_{mC} are respectively the concentrations and K_m of the substrates PEP, AMP and PP_i; P, Q and R and K_{mP}, K_{mQ} and K_{mR} are the concentrations and K_m of the products Pyr, ATP, P_i; $\alpha|V_f - V_{f0}|$ is the adjustment term with α, a defined number, V_{f0}, PPDK maximum rate in the forward direction used in the *in vitro* reconstitution and V_f is PPDK maximum rate in the forward direction in the model.

Enzyme	Kinetic equations
**PGAM**	$v = \dfrac{V_f \frac{[3PG]}{K_{m3PG}} - V_r \frac{[2PG]}{K_{m2PG}}}{1 + \frac{[3PG]}{K_{m3PG}} + \frac{[2PG]}{K_{m2PG}} + \frac{[PP_i]}{K_{iPP_i}}}$
**ENO**	$v = \dfrac{V_f \frac{[2PG]}{K_{m2PG}} - V_r \frac{[PEP]}{K_{mPEP}}}{1 + \frac{[2PG]}{K_{m2PG}} + \frac{[PEP]}{K_{mPEP}} + \frac{[PP_i]}{K_{iPP_i}} + \frac{[3PG]}{K_{i3PG}}}$
**PPDK**[a]	$v = \dfrac{V_f \left(ABC - \frac{PQR}{K_{eq}}\right)}{K_{mA}B + K_{mB}A + K_{mC}B + K_{mB}C + \frac{V_f K_{mQ} P}{V_r K_{eq}} + \frac{V_f K_{mP} Q}{V_r K_{eq}} + \frac{V_f K_{mQ} R}{V_r K_{eq}} + \frac{V_f K_{mR} Q}{V_r K_{eq}} + ABC + \frac{V_f PQR}{V_r K_{eq}} + \alpha\left|V_f - V_{f0}\right|}$

Supplementary Table 2. Description of the new generated dataset (Dataset 1).

	PGAM	ENO	PPDK	J_{pred}
**Data count**	68,950	68,950	68,950	68,950
**Mean**	499.82	499.92	499.87	83.46
**Standard deviation**	295.87	295.78	295.82	55.32
**Minimum value**	0	0	0	0
**25%**	250	250	250	35.998
**50%**	500	500	500	80.23
**75%**	750	750	750	127.6
**Maximum value**	1,000.0	1,000.0	1,000.0	215.45

Supplementary Table 3. Summary table of statistical measurements for each predictive model for **Supplementary Table 6** (2,000 data).

	Training set		Test set	
**Model**	**cvRMSE**	**cvR^2**	**RMSE**	**R^2**
**XGBoost Linear**	0.833	0.995	0.051	1

**QRF (RF)**	0.931	0.994	0.076	1
**Cubist**	0.49	0.998	0.28	1
**Rborist (RF)**	0.883	0.995	0.291	1
**ANN**	0.666	0.997	0.637	0.998
SVM Radial	1.404	0.988	1.053	0.994
SVM Poly	1.454	0.986	1.282	0.991
bagEarth GCV (bagging MARS)	3.461	0.92	3.248	0.941
Ridge	6.038	0.747	5.882	0.8
Bayesian GLM	6.038	0.747	5.882	0.8
Spike-and-slab	6.038	0.747	5.882	0.8
Lasso	6.106	0.747	5.969	0.802
PLS	8.601	0.494	9.074	0.521

Supplementary Table 4. Description of the new generated dataset (Dataset 2).

	**TryR**	**TXN**	**TXNPx**	J_{pred}
**Data count**	1,671	1,671	1,671	1,671
**Mean**	203.88	69.84	161.09	6.6
**Standard deviation**	79.8	27.7	37.15	3.25
**Minimum value**	16.1	0	57.6	0
**25%**	143.75	52.95	151.7	3.82
**50%**	264	88	179	7.58
**75%**	264	88	179	9.58
**Maximum value**	264	102	220	11.46

Supplementary Table 5. Description of the new generated dataset (Dataset 3).

	**Time (h)**	**Oil flow (L/h)**	**Aeration rate (L/h)**	**Vessel weight (kg)**	**Carbon evolution rate (g/h)**	**Vessel volume (L)**	**CO_2 in off-gas (%)**	**Penicillin concentration (g/L)**
**Data count**	113,935	113,935	113,935	113,935	113,935	113,935	113,935	113,935
**Mean**	114.75	26.35	65.25	81,076.73	1.25	73,312.87	1.44	14.33
**Standard deviation**	66.99	4.95	11.69	10,097.23	0.48	8,599.64	0.5	9.93
**Minimum value**	0.2	22	20	60,395	0.029	56,549	0.075	0
**25%**	57	23	60	73,018.5	0.98	65,885.5	1.23	5.53
**50%**	114	23	65	84,367	1.40	75,770	1.6	14.38
**75%**	171	30	75	88,608	1.62	79,892	1.76	22.69
**Maximum value**	290	35	75	107,010	2.05	95,716	7.12	36.18

Supplementary Table 6. Dataset of 2,000 simulated enzyme activity ratios and their corresponding pathway flux (*J*). See .xlsx file (Table1.xlsx).

Supplementary Table 7. Dataset 1 of experimental and simulated enzyme activity ratios and their corresponding pathway flux (*J*). See .xlsx file (Table2.xlsx).

Supplementary Table 8. Dataset 2 of experimental and simulated enzyme activity ratios and their corresponding pathway flux (*J*). See .xlsx file (Table3.xlsx).

Supplementary Table 9. Dataset 3 of experimental recordings of the process of penicillin fermentation in a bioreactor. See .xlsx file (Table4.xlsx).
